# Combined analysis of metagenome and transcriptome revealed the adaptive mechanism of different golden Camellia species in karst regions

**DOI:** 10.3389/fpls.2023.1180472

**Published:** 2023-11-20

**Authors:** Jianxiu Liu, Haidu Jiang, Yang Huang, Lisha Zhong, Qin Xu, Quanguang Yang, Shengyuan Liu, Xiao Wei, Yu Liang, Shengfeng Chai

**Affiliations:** ^1^ Guangxi Key Laboratory of Plant Functional Phytochemicals and Sustainable Utilization, Guangxi Institute of Botany, The Chinese Academy of Sciences, Guilin, China; ^2^ Key Laboratory of Ecology of Rare and Endangered Species and Environmental Protection, Guangxi Key Laboratory of Landscape Resources Conservation and Sustainable Utilization in Lijiang River Basin, College of Life Science, Guangxi Normal University, Guilin, China; ^3^ School of Mechanical and Electrical Engineering, Guilin University of Electronic Technology, Guilin, China; ^4^ Golden Camellia National Nature Reserve Management Center, Fangchenggang, China; ^5^ Administration of Nonggang National Nature Reserve of Guangxi, Chongzuo, China

**Keywords:** golden Camellia, adaptation mechanism, karst regions, metagenome, transcriptome

## Abstract

*Camellia* sect. *Chrysantha* is an important rare and protected plant species. Some golden Camellia species grow in karst soil while others grow in acidic soil. In order to study the adaptation mechanism of golden Camellia to the karst environment, four species of golden Camellia growing in the karst soil (*Camellia pubipetala*, *Camellia perpetua*, *Camellia grandis*, and *Camellia limonia*) and four species growing in the acidic soil (*Camellia nitidissima*, *Camellia euphlebia*, *Camellia tunghinensis*, and *Camellia parvipetala*) were selected for this study. Combining the metagenome and transcriptome, the structure and function of the rhizosphere microbial communities and the gene expression in roots of golden Camellia were analyzed. The results showed that the rhizosphere microbial communities in different golden Camellia were significantly different in abundance of Acidobacteria, Actinobacteria, Candidatus_Rokubacteria, Nitrospirae, Planctomycetes, and Candidatus_Tectomicrobia. The proportion of Candidatus_Rokubacteria was significantly higher in the rhizosphere soil of four species of golden Camellia grown in karst areas, compared to *C. nitidissima*, *C. euphlebia*, and *C. tunghinensis*. The linear discriminant analysis Effect Size showed that *C. parvipetala* was similar to karst species in the enrichment of ABC transporters and quorum sensing. During the transcriptome analysis, numerous upregulated genes in four karst species, including *CYP81E*, *CHS*, *F3H*, *C12RT1*, *NAS*, and *CAD*, were found to be enriched in the secondary metabolite synthesis pathway in the KEGG library, when compared to *C. tunghinensis*. This study provides information for plant adaptation mechanisms on the rhizosphere soil microbial composition and gene expression in secondary metabolic pathways to karst habitats and its distribution in karst areas.

## 1 Introduction

*Camellia* sect. *Chrysantha* Chang is an evergreen shrub or small tree of the genus *Camellia* and the family Theaceae (Wei et al., 2017). They are second-class protected wild plants in China. At present, there are more than 20 species of plants in the *Camellia* sect. *Chrysantha* reported, and it is famous for its golden Camellia flowers. According to the different ecological environments and growth soils of golden Camellia, it is mainly divided into acid soil and calcareous soil golden Camellia (Su and Mo, 1988). Acid soil golden Camellia mainly grows in acidic soil, the soil property is mainly acid red soil, and calcareous soil golden Camellia mainly grows in karst soil. Different from the acidic soil, the soil in karst regions is alkaline, with high content of calcium and magnesium, low soil content, and less water (Zhu et al., 2022a). Therefore, high Ca^2+^ and drought stress become the main factors limiting plant growth in karst regions. So far, no species of golden Camellia has been found to be able to grow in both acidic and calcareous soil areas. Thus, the adaptation mechanism of golden Camellia to the environment may be species-specific, leading to different environmental adaptation mechanisms among different species of golden Camellia. Therefore, it is necessary to compare the species growing in acidic soil with the ones growing in karst soil to investigate the adaptation mechanism of golden Camellia.

At present, some studies have investigated the adaptation of plants in karst regions, and offered some basic information for karst ecosystem and vegetation protection. Firstly, the water stress in karst regions is an important factor for plant growth. It has been found that the physiological morphology of plants has an important drought resistance. Plants in karst regions often have a thick cuticle by closing epidermal stomata, reducing water evaporation and transpiration (Deng et al., 2004; Rong et al., 2005). Physiologically, karst plants can alleviate the damage caused by drought stress through the accumulation of osmoregulatory substances, including soluble protein, proline, and flavonoids, to enhance their drought resistance (Guo et al., 2018). Secondly, in addition to drought, high calcium is also one of the significant characteristics of soil in karst regions. As an essential element for plant growth and development, calcium plays an important role in signal transduction, but high content of calcium in soil will cause osmotic damage to plant growth (Wang et al., 2011; Li et al., 2014). In order to adapt to the high calcium environment in karst regions, plants have strong regulation of low intracellular calcium content. It was found that plants in karst regions could reduce the effects of Ca^2+^ on their growth and development by transporting Ca^2+^ to other parts of the body (Jarrett et al., 1982; Dolman et al., 2003; Zhang et al., 2017; Jin et al., 2018).

Certain types of golden Camellia growing in karst regions exhibit some adaptive features to their karst environment. In our previous study, golden Camellia from different growth areas (*Camellia nitidissima* from acidic soil and *Camellia limonia* from karst soil) were treated with high calcium to analyze the adaptation mechanism of golden Camellia in karst environment according to their differences in morphology and physiology, and it was found that golden Camellia growing in the karst soil has a more stable photosynthetic capacity and water retention ability (Liu et al., 2022a). Various studies have recently demonstrated that golden Camellia was capable of adapting to drought stress in karst areas by changing the size of its stomata and by developing leathery leaves (Chai et al., 2021; Zhu et al., 2021; Zhu et al., 2022b), but the specific adaptation mechanism in transcription level is still unclear. Moreover, soil microorganisms in different regions are also crucial to plant growth, and there are few studies that focus on the roles of rhizosphere microorganisms in the adaptation mechanism of karst plants.

In this study, a total of eight species of golden Camellia (four in acidic soil and four in karst soil) were used. The structural and functional characteristics of rhizosphere microorganisms in eight different species of golden Camellia growth regions were analyzed by metagenomic analysis, and the roles of rhizosphere microorganisms in the adaptation mechanism of golden camellia were investigated. In addition, regulator genes in plant roots were analyzed by transcriptome. The combination of metagenome and transcriptome was performed to investigate the adaptive mechanisms of golden Camellia to karst regions. This result provides valuable insights into the molecular level of adaptive mechanisms and gene functions in karst plants.

## 2 Materials and methods

### 2.1 Plant and rhizosphere soil sample collection

**Figure 1:**
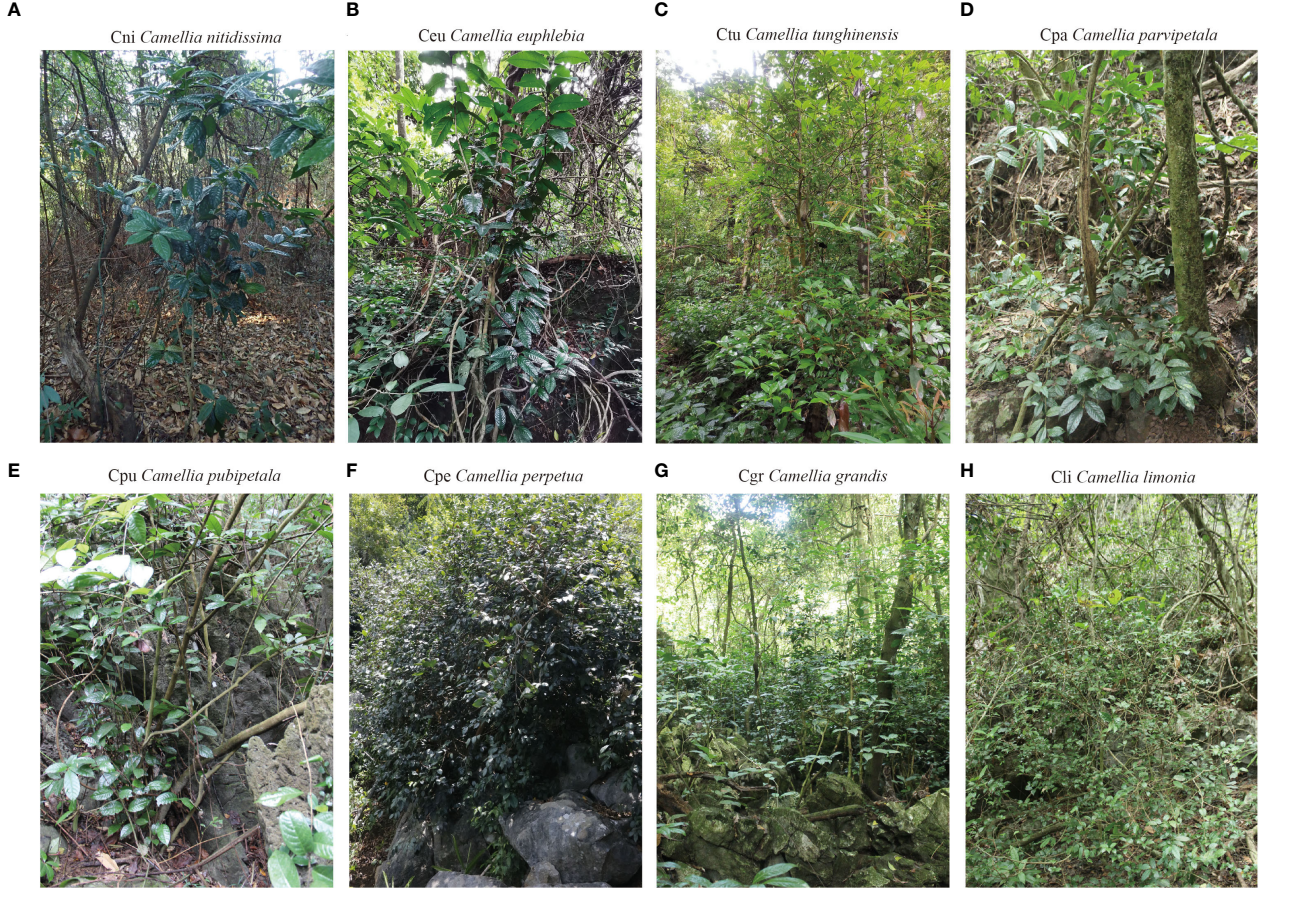
Photographs of the habitats of different golden Camellia growing in normal or karst soil. **(A–D)** *Camellia nitidissima* (Cni), *Camellia euphlebia* (Ceu), *Camellia tunghinensis* (Ctu), and *Camellia parvipetala* (Cpa) grown in acidic soil, respectively. **(E–H)** *Camellia pubipetala* (Cpu), *Camellia perpetua* (Cpe), *Camellia grandis* (Cgr), and *Camellia limonia* (Cli) grown in karst soil.

In this study, the root and rhizosphere soil samples of eight species of golden Camellia that grow in the karst soil or acidic soil were used as experimental materials in different nature reserves in Guangxi Zhuang Autonomous Region, China. Four species growing in acidic soil were *C. nitidissima* (Cni), *Camellia euphlebia* (Ceu), *Camellia tunghinensis* (Ctu), and *Camellia parvipetala* (Cpa), and four species growing in karst soil were *Camellia pubipetala* (Cpu), *Camellia perpetua* (Cpe), *Camellia grandis* (Cgr), and *Camellia limonia* (Cli) (details in **Table 1**; **Figure 1**). Three biological replicates were taken for the rhizosphere soil and root samples of each golden Camellia species. A total of 24 rhizosphere soil and 24 root samples were collected (**Figure S1**). The rhizosphere soil collection included washing the roots with distilled water three to six times to completely remove non-rhizosphere soil. About 0.5 g of rhizosphere soil, which refers to the soil within a few millimeters of the root surface of the golden Camellia, was collected and packed in sterile centrifuge tubes. Root samples were taken from the root tip, meristematic, elongation, and maturation zones, respectively. Prior to DNA and RNA extraction, the samples were frozen in liquid nitrogen and transported to the laboratory on dry ice. The roots of golden Camellia were ultrasonically washed twice with sterile water for 5 min each time to remove soil and microorganisms from the root surface. After washing, approximately 0.5 g of plant root tissue was selected as root samples and collected in a sterile 1.5-mL centrifuge tube for RNA extraction. The soil pH and calcium concentration were determined and published as part of the results (**Supplementary Table S1**) (Zhu et al., 2022a).

**Table 1:** Sampling information of eight species golden Camellia.

Species	Habitat soil type	Sampling location
*Camellia nitidissima* CNI	Acidic soil	Fangcheng National Nature Reserve of Guangxi (21°44'51" N, 108°6'39" E)
*Camellia euphlebia* CEU		Fangcheng National Nature Reserve of Guangxi (21°45'27" N, 108°5'53" E)
*Camellia tunghinensis* CTU		Fangcheng National Nature Reserve of Guangxi (21°45'6" N, 108°3'24" E)
*Camellia parvipetala* CPA		Paihe Village, Zhilang Township, ningming County (21°33'18" N, 106°48'20" E)
*Camellia pubipetala* CPU	Calcareous soil (karst soil)	Longhushan Nature Reserve of Guangxi (22°57'39"N, 107°37' 25"E)
*Camellia perpetua* CPE		Pairu Village,Jiangzho District, Chongzuo City, Cuangxi (22°34'52" N, 107°25'23" E)
*Camellia grandis* CGR		Nonggang National Nature Reserve of Guangxi (22°28'12" N, 106°54'46" E)
*Camellia limonia* CLI		Nonggang National Nature Reserve of Guangxi (22°14'29" N, 107°3'31" E)

### 2.2 Metagenome sequencing and analysis

#### 2.2.1 DNA extraction, library construction, and sequencing

The E.Z.N.A.^®^Soil DNA Kit from Omega Bio-Tek (GA, USA) was utilized to extract DNA from soil samples. Following DNA extraction, the purity and concentration of the DNA and the length of extracted DNA were measured with NanoDrop2000 from Thermo Fisher Scientific (MA, USA) and using 1% agarose gel electrophoresis. DNA fragmentation was accomplished using Covaris M220 from Shanghai Pudi Biotechnology Co., Ltd. (Shanghai, China). Primers 515F (5′-barcode-GTGCCAGCMGCCGCGCGGGTAA-3′)/806R(5′-GGACTACHVGGGTWTCTAAT-3′) and ITS1F (5′-barcode-CTTGGGTCATTTA GAGGAAGTAA-3′)/ITS2R (5′-GCTGCGTTCTTCATCGATGC-3′) were used to amplify the V3–V4 region of the bacterial 16S rRNA gene and the ITS1 gene region of fungi, respectively. Amplicons were detected on 2% (w/v) agarose gels and purified using the AxyPrep DNA Gel Extraction Kit (Axygen Biosciences). PE libraries were constructed using the NEXTFLEX^®^ Rapid DNA-Seq Kit (Bioo Scientific, Texas, USA). The construction process included the following: (1) connecting the joints, (2) removing the joint self-connecting segments using magnetic bead screening, (3) enriching the library templates by PCR amplification, and (4) recovering the library through magnetic beads from the PCR product. The genome sequencing was performed with Illumina Novaseq 6000 (Illumina, CA, USA) in Shanghai Megi Biomedical Technology Co.

#### 2.2.2 Sequence pre-processing, assembly, gene set construction, and abundance calculation

Fastp version 0.23.0 was used for data quality control based on the original sequencing data. Low-quality and N-containing reads were removed to obtain high-quality sequences needed for subsequent analysis. After quality control, the short segment sequence was assembled, and the open reading frames were predicted. After quality control, Megahit was used to assemble multiple short sequences that were mixed together. (Megahit can be found at https://github.com/voutcn/megahit). Sequences with gene sequence length >100 bp were predicted for ORFs using Prodigal (http://compbio.ornl.gov/prodigal). The predicted gene sequences of the samples were clustered, the non-redundant gene set was constructed, and the base sequence of the non-redundant gene set genes was obtained using CD-HIT (http://www.bioinformatics.org/cd-hit/). SOAPaligner (http://soap.genomics.org.cn/) was used to assess the quality of reads for each sample, compare the redundant gene sets, and determine the abundance of genes present in the statistical sample. Gene abundance was quantified as Reads Per Kilobase Million (RPKM).

#### 2.2.3 Annotations on species and functions

Non-redundant gene sets from various databases, including NR database, carbohydrate-active enzyme database, and Kyoto Encyclopedia of Genes and Genomes (http://www.genome.jp/kegg/) database, were compared using DIAMOND (https://github.com/bbuchfink/diamond) with the blastp parameter and an E-value of ≤1e-5.

### 2.3 Transcriptome sequencing and sequence analysis

#### 2.3.1 RNA extraction, library construction and sequencing

Total RNA was extracted from the root samples using Plant RNA Purification Reagent (Invitrogen, Carlsbad, CA, USA). RNA concentration and purity were detected using Nanodrop2000 (Thermo Fisher Scientific, MA, USA), while RNA integrity was verified via 1% agarose gel electrophoresis. Then, 5 μg of total RNA from each sample was used for library preparation with a TruSeq Stranded Total RNA Sample Preparation Kit (Illumina, San Diego, USA). RIN value was determined using Agilent 2100 (Agilent Technologies, Palo Alto, CA, USA). The fragment RNA was then reversely transcribed into cDNA, then connected to adaptor, and the original data were sequenced on Illumina Novaseq 6000 (Illumina, CA, USA).

#### 2.3.2 Data preprocessing, comparison, and expression analysis

Clean reads were obtained by removing raw reads from the original data for subsequent analysis. Clean data (reads) after quality control were used for TopHat2 (http://tophat.cbcb.umd.edu/) software comparison with reference genome (*Camellia sinensis*, https://www.ncbi.nih.gov/genome/?term=GCF_004153795.1) to get mapped data (reads). Cufflinks (http://cole-trapnelllab.Making.IO/Cufflinks/) was used to assemble the mapped reads splicing. After genes were identified in the gene ontology (GO) and KEGG database, the information of the function of genes and transcription was obtained. RSEM (http://deweylab.biostat.wisc.edu/rsem/) was used to quantitatively analyze the gene expression levels, and the quantitative index was the transcripts per million reads (TPM). DESeq2 was used for inter-sample or inter-group gene differential expression analysis, and the differentially expressed genes (DEGs) were identified. In this study, genes and transcripts with absolute multiple change ≥2 and false discovery rate (FDR) < 0.05 were DEGs.

### 2.4 Statistical analysis

TBtools (version 1.047) was used for heat map analysis of DEGs. The Wilcoxon rank sum test was used to detect significant differences in gene abundance in soil samples in metagenome data, and the mean was analyzed at the *p* < 0.05 significance level.

## 3 Results

### 3.1 Metagenome and transcriptome datasets of eight species of golden Camellia growing in karst soil and acidic soil

After quality control, the metagenomic data yielded approximately 116 million clean reads, averaging approximately 5 million clean reads per sample. In each sample, clean reads comprised over 98% of the raw reads. About 1 million contigs were obtained with an average of 443,771 contigs per sample. The high quality of sequence assembly was reflected in N50 and N90 values. Prediction of open reading frames (ORFs) yielded about 1.2 million sequences, ranging from 102 to 9,627 bp. The characteristics of the DNA-sequencing results indicate that the data are of high quality and suitable for further analysis (**Supplementary Table S2**).

Similarly, for the transcriptome data after quality control, a total of 165.05 Gb clean data was obtained, and the clean data of each sample reached more than 5.87 Gb, the percentage of Q30 base was more than 91.64%, and the error rate of each sample was about 0.2%. GC content ranged from 44.08% to 47.64%, which met the requirements of subsequent analysis. The clean reads of each sample were aligned with the designated reference genome, and the alignment rate ranged from 68.02% to 76.58% (**Supplementary Table S3**). At the same time, the gene expression level of each sample was in line with the sequencing precision range of transcriptome, and the data were evenly distributed (**Figure S2**).

### 3.2 Characteristics of rhizosphere microbial communities of golden Camellia growing in the acidic soil and karst soil

**Figure 2:**
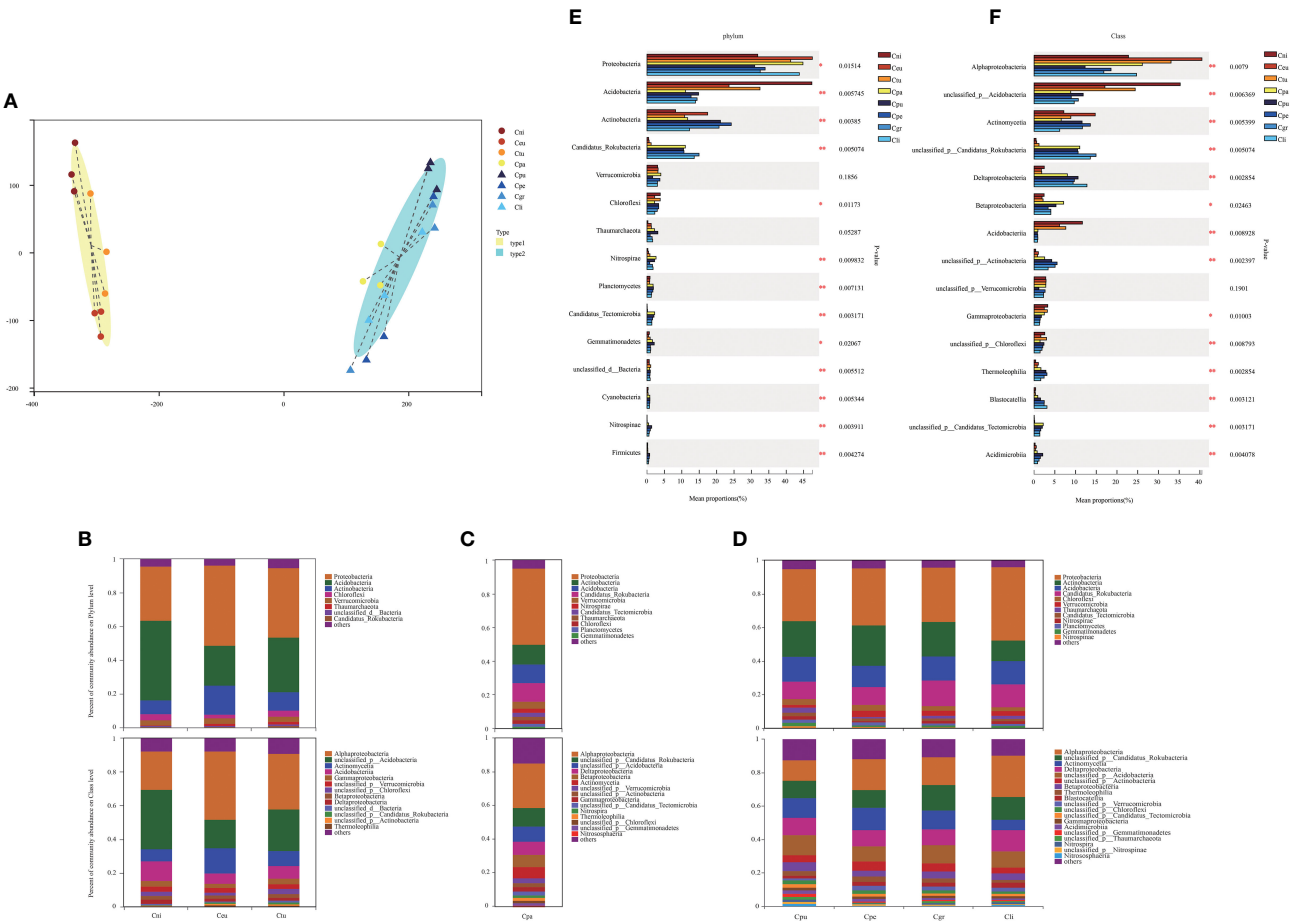
Characteristics of rhizosphere microbial communities of golden Camellia growing in normal or karst soil. **(A)** Typing analysis for all soil samples based on the genus level of rhizosphere microorganisms and the distance algorithm is JSD. **(B–D)** The relative abundance of microorganisms in eight golden Camellia species for major taxa at phylum (upper) and class level (lower). **(E, F)** The microbial abundance at the phylum and class level in eight golden Camellia species. Star means *p*-value ≤ 0.05.

There are many important factors that affect the growth of golden Camellia in specific habitats (**Figure 1**). Not only in terms of phenotype, but also the characteristics of soil microbial community are closely related to the habitat adaptation of golden Camellia. Based on metagenome analysis, the characteristics of rhizosphere microbial community structure of different golden Camellia growing in the acidic soil or karst soil were examined, and their roles were analyzed. **Figure 2A** shows that the distances of the three biological replicates of the same golden Camellia were close, indicating the reliability of metagenome sequencing data. The soil microbial community structure of golden Camellia (Cni, Ceu, and Ctu) in the same habitat (acidic soil) has a similarity. Moreover, the rhizosphere microbial community structure of golden Camellia (Cpu, Cpe, Cgr, and Cli) growing in karst soil was also similar. It is worth noting that Cpa growing in the acidic soil was closer to the karst soil in terms of community structure.

Based on the genes, the species classification annotation of eight golden Camellia species of rhizosphere microorganisms was statistically analyzed (**Table 2**). In acidic soil (Cni, Ceu, Ctu, and Cpa), the number of microbial phyla ranges from 200 to 209, and the number of microbial species ranges from 16,714 to 18,208. In karst soil (Cpu, Cpe, Cgr, and Cli), the number of microbial phyla varied between 208 and 217 and the number of microbial species varied between 19,454 and 21,917. These results demonstrated that the species diversity of rhizosphere microorganisms in different habitats was different, and the diversity of rhizosphere microorganisms in karst soil was higher than that in acidic soil.

**Table 2:** The information of species classification and annotation in rhizosphere microorganisms of 8 species golden Camellia.

Groups	Domain	Kingdom	Phylum	Class	Order	Family	Genus	Species
CNI	5	10	200	364	675	1241	3420	16714
CEU	5	11	202	369	686	1302	3589	17472
CTU	5	10	209	380	686	1269	3617	18208
CPA	5	12	205	361	650	1195	3564	17733
CPU	5	10	208	378	693	1283	3786	19454
CPE	5	12	217	398	746	1405	4126	21917
CGR	5	12	214	381	706	1332	4007	21458
CLI	5	11	211	379	688	1256	3730	19809

The rhizosphere microbial composition of eight golden Camellia species based on phylum and class levels was analyzed (**Figures 2B–D**). At the phylum level, the top three phyla with higher abundance for eight golden Camellia species were all Proteobacteria, Actinobacteria, and Acidobacteria. However, the mean proportions of microbial phyla in different golden Camellia were highly significantly different, particularly in Acidobacteria, Actinobacteria, Candidatus_Rokubacteria, Nitrospirae, Planctomycetes, and Candidatus_Tectomicrobia (0.001< *p*-value ≤ 0.01) (**Figure 2E**). Among the top five microbial phyla in abundance, only Acidobacteria was significantly more abundant in normal species (Cni, Ceu, and Ctu) than in Cpa, Cpu, Cpe, Cgr, and Cli. The abundance of Candidatus_Rokubacteria was significantly higher in Cpa, Cpu, Cpe, Cgr, and Cli than in Cni, Ceu, and Ctu. This result indicated that mainly Acidobacteria and Candidatus_Rokubacteria have significant abundance differences between rhizosphere soil of normal and karst soils. Furthermore, the mean proportions of Acidobacteria and Actinobacteria also significantly differed in Cni, Ceu, and Ctu growing in acidic soil, among which the mean proportion of Acidobacteria in Cni was the highest (47.36%), and the mean proportion of Acidobacteria in Ceu was the lowest (23.56%). Among Actinobacteria, Ceu was the highest (17.41%) and Cni was the lowest (8.214%) (**Figure S3A**). Nevertheless, there were no significant differences in microbial phyla in karst soil, except for Nitrospirae which had low mean proportions (**Figure S3C**).

At the class level, Alphaproteobacteria was the most dominant bacteria in the eight golden Camellia species (**Figures 2B–D**). Besides Alphaproteobacteria, the dominant bacteria include unclassified_p_Acidobacteria, Actinomycetia, Acidobacteriia and Gammaproteobacterial in Cni, Ceu, and Ctu. Unclassified_p_Candidatus_Rokubacteria, unclassified_p_Acidobacteria, Deltaproteobacteria, Betaproteobacteria, and Actinomycetia were dominant bacterial community in Cpa. Unclassified_p_Candidatus_Rokubacteria, Actinomycetia, Deltaproteobacteria, unclassified_p_Acidobacteria, unclassified_p_Actinobacteria, Betaproteobacteria, and Thermoleophilia were dominant bacterial community in karst species (Cpu, Cpe, Cgr, and Cli). The mean proportions of Alphaproteobacteria, Deltaproteobacteria, Betaproteobacteria, and Gammaproteobacterial differed significantly in eight golden Camellia species (**Figure 2F**). The mean proportions of Alphaproteobacterial ranged from 22.77% (Cni) to 40.49% (Ceu) in acidic soil, and it ranged from 12.3% (Cpu) to 24.72% (Cli) in karst soil. However, the proportions of Deltaproteobacteria and Betaproteobacteria in karst soil were significantly higher than those in acidic soil (Cni, Ceu, Ctu). Only the proportion of Alphaproteobacteria was different in the same habitat (karst soil) (**Figures S3B, D**). At phylum and class levels, the differences in microbial community structure between the same or different habitats may reflect the functional characteristics of soil microorganisms.

### 3.3 Functional pathways of rhizosphere microorganisms of golden Camellia growing in the normal and karst soils

**Figure 3:**
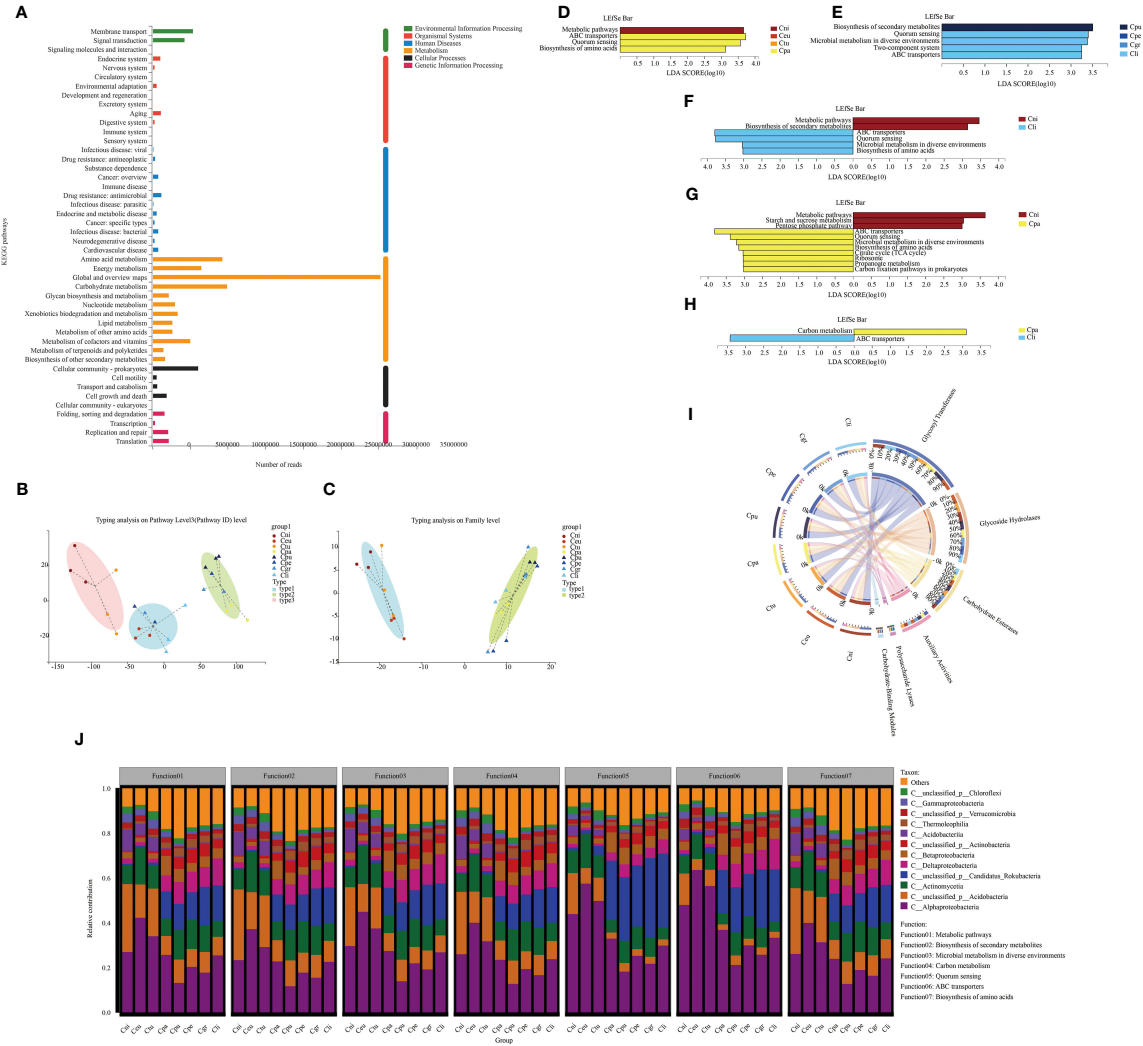
Functional pathways of rhizosphere microorganisms of golden Camellia growing in the normal and karst soils. **(A)** KEGG functional annotation for all soil samples was based on pathway levels 1 and 2. The abundance was calculated as Reads Number. **(B, C)** Typing analysis for rhizosphere microbial gene functions was based on KEGG **(B)** and CAZy **(C)** databases. **(D–H)** LEfSe analysis at KEGG pathway level 3 with significant differences in rhizosphere microbial gene function. Cni, Ceu, Ctu, and Cpa **(D)**. Cpu, Cpe, Cgr, and Cli **(E)**. Cni and Cli **(F)**. Cni and Cpa **(G)**. Cpa and Cli **(H)**. The threshold is 3. **(I)** The functional composition of rhizosphere microbial community was based on the CAZy database. **(J)** Functional contribution analysis of the top seven functions at KEGG pathway level 3 based on the abundance of rhizosphere microbial community at the class level.

The main functional pathways of rhizosphere microorganisms could have a certain impact on plant growth. Therefore, the functional pathways of soil microorganisms were analyzed. The functional annotation of non-redundant genes in all samples was performed to compare the abundance of functional genes in the rhizosphere microorganisms of eight golden Camellia species based on the Kyoto Encyclopedia of Genes and Genomes (KEGG) database and carbohydrate-active enzyme (CAZy) functional database. In total, there were 111,555,026 KEGG pathway-associated genes (**Supplementary Table S4**) and 6,721,670 CAZy genes (**Supplementary Table S5**) from all rhizosphere soil samples. The global and overview maps, the carbohydrate metabolism, amino acid metabolism, and energy metabolism were the main functional pathways based on pathway level 2 (**Figure 3A**). Based on the KEGG pathway level 3, all soil samples were divided into three main types (1: Cni and Ctu; 2: Ceu, Cpe, and Cli; 3: Cpa, Cpu, and Cgr) (**Figure 3B**). Those were divided into two types based on CAZy (1: Cni, Ceu, and Ctu; 2: Cpa, Cpu, Cpe, Cgr, and Cli) (**Figure 3C**). This reflected that the functions of soil microorganisms of the same type were somewhat similar and Cpa was special with more similarity with karst species.

The LEfSe analysis was performed on all KEGG pathway level 3 to identify pathways with significant differences in non-redundant gene sets after KEGG annotation (**Figures 3D–H**). In normal species (Cni, Ceu, Ctu, and Cpa), Cni was mainly related to the metabolic pathway, while Cpa was mainly related to ABC transporters, quorum sensing, and biosynthesis of amino acids. However, no significantly different pathways were identified in Ceu and Ctu (**Figure 3D**). In addition, in karst species (Cpu, Cpe, Cgr, and Cli), Cpu was mainly related to biosynthesis of secondary metabolites. Cli was mainly related to quorum sensing, microbial metabolism in diverse environments, two-component system, and ABC transporters. Similarly, no significantly different pathways were identified between Cpe and Cgr (**Figure 3E**). When Cni (normal species) was compared with Cpu, Cpe, Cgr, and Cli, respectively, Cni was associated with the metabolic pathway in each comparison. Cpu, Cpe, Cgr, and Cli were also associated with ABC transporters and quorum sensing (**Figure 3F**; **Figure S3A–C**). These results indicated that soil microbial functions in karst soil were mainly related to ABC transporters and quorum sensing. Moreover, normal species were mainly related to the metabolic pathway. Moreover, Cpa was also associated with ABC transporters and quorum sensing in the comparison between Cni and Cpa, suggesting that microbial functions in Cpa shared some common features with those in golden Camellia growing in karst soil (**Figure 3G**). Also, Cpa was only related to carbon metabolism, while Cli was only related to ABC transporters in the comparison between Cpa and Cli (**Figure 3H**).

The non-redundant gene set obtained six classes after annotation in the CAZy functional database, among which glycosyl transferases had the highest proportion (Cni: 37%; Ceu: 35%; Ctu: 35%; Cpa: 35%; Cpu: 35%; Cpe: 36%; Cgr: 36%; Cli: 37%), followed by the glycoside hydrolases (Cni: 28%; Ceu: 29%; Ctu: 29%; Cpa: 27%; Cpu: 27%; Cpe: 27%; Cgr: 27%; Cli: 25%), carbohydrate esterases (Cni: 17%; Ceu: 19%; Ctu: 19%; Cpa: 20%; Cpu: 19%; Cpe: 18%; Cgr: 19%; Cli: 20%), auxiliary activities (Cni: 12%; Ceu: 12%; Ctu: 12%; Cpa: 13%; Cpu: 13%; Cpe: 13%; Cgr: 13%; Cli: 12%), polysaccharide lyases (Cni: 2.9%; Ceu: 2.2%; Ctu: 2.6%; Cpa: 2.7%; Cpu: 3.2%; Cpe: 3.0%; Cgr: 3.2%; Cli: 3.0%), and carbohydrate-binding modules (Cni: 2.4%; Ceu: 2.4%; Ctu: 2.4%; Cpa: 3.0%; Cpu: 3.1%; Cpe: 3.0%; Cgr: 2.8%; Cli: 2.7%) (**Figure 3I**).

### 3.4 The correlations between rhizosphere microbial community structure and function in normal and karst soils

The function of rhizosphere microorganisms under different golden Camellia is related to its composition. Species and functional contribution analysis further confirmed the relationship between rhizosphere microorganisms at the class level and KEGG pathway level 3 (**Figure 3J**). In normal species Cni, Ceu, and Ctu, the top four contributing microbial classes in the metabolic pathway were Alphaproteobacterial (43.56%–28.47%), Actinomycetia (17.34%–9.11%), unclassified_p_Acidobacteria (20.26%–9.23%), and Acidobacteriia (17.93%–9.04%). Therefore, the high abundance of Alphaproteobacteria, unclassified_p_Acidobacteria, and Acidobacteriia in Cni, Ceu, and Ctu resulted in a higher abundance of metabolic pathway than Cpa, Cpu, Cpe, Cgr, and Cli. Quorum sensing and ABC transporters were mainly related to rhizosphere microorganisms in karst soil. Alphaproteobacteria (33.84%–22.38%), Actinomycetia (12.70%–7.03%), and unclassified_p_Candidatus_Rokubacteria (31.02%–23.77%) were the major contributing classes to quorum sensing. The ABC transporters pathway was mainly associated with Alphaproteobacteria (37.19%–25.37%), unclassified_p_Candidatus_Rokubacteria (22.33%–15.96%), and Deltaproteobacteria (14.93%–9.84%). Overall, the functional contribution patterns of soil microorganisms in Cpa were similar to those in golden Camellia growing in karst soil.

### 3.5 The correlations of root gene expression among the eight species of golden Camellia

**Figure 4:**
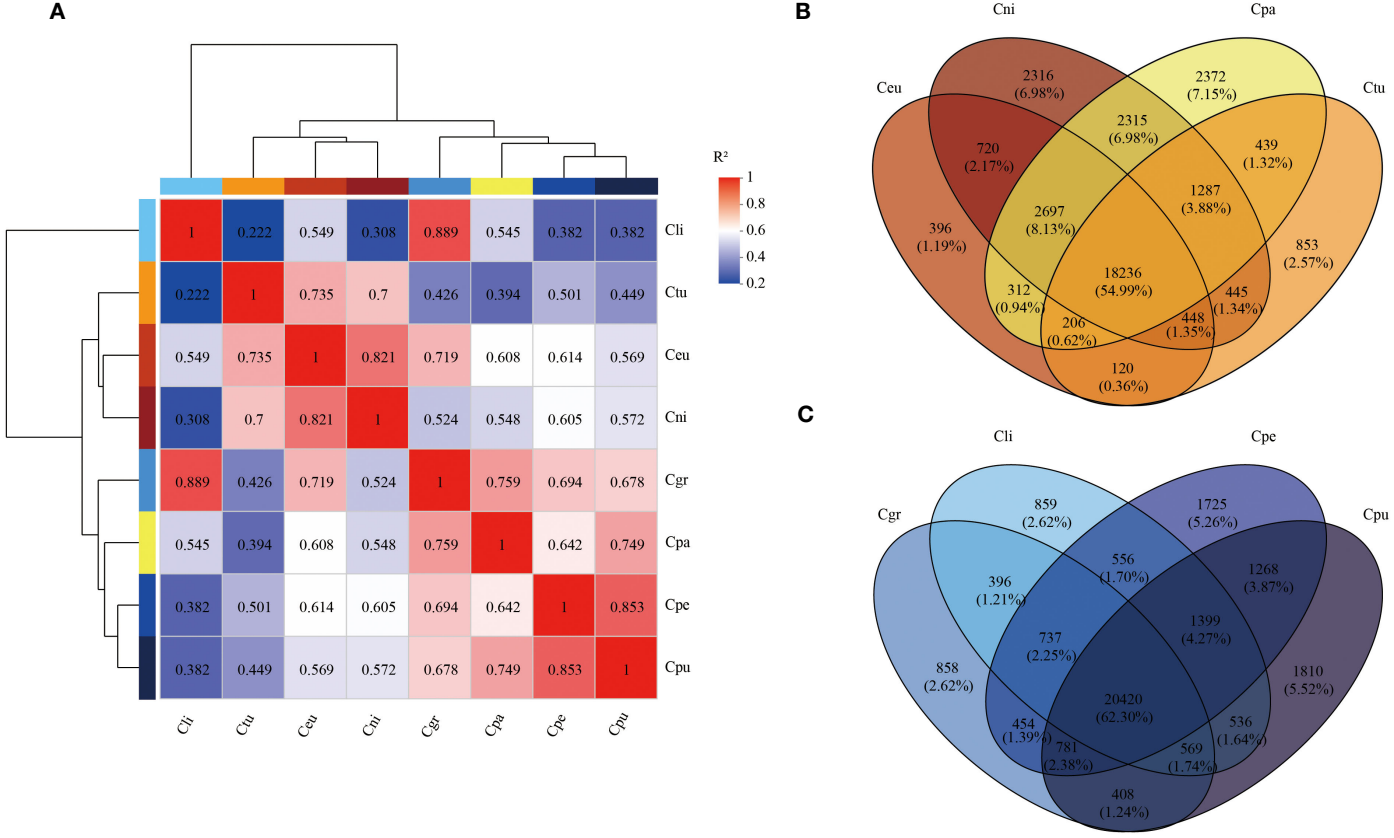
Transcriptome sequencing analysis of root samples of golden Camellia. **(A)** Correlation analysis of eight golden Camellia species. **(B)** The Venn diagram of the root genes at Cni, Ceu, Ctu, and Cpa. **(C)** The Venn diagram of the root genes at Cpu, Cpe, Cgr, and Cli.

The correlation of root gene expression of eight species of golden Camellia was performed (**Figure 4A**). Cpa was semblable to that of Cpu (0.749) and Cgr (0.759). There was comparability in Cni, Ceu, and Ctu because the correlations among Cni, Ceu, and Ctu were all above 0.7. Furthermore, the correlation among golden Camellia growing in karst soil was also above 0.678. In particular, Cli and Cgr showed a high correlation of 0.889.

In Cni, Ceu, Ctu, and Cpa, there were 18,236 (54.99%) common genes, and Cpa had the highest number of unique genes (2,372), which was consistent with the metagenomic results (**Figure 4B**). Among Cpu, Cpe, Cgr, and Cli growing in karst soil, there were 20,420 (62.30%) common genes, which might be related to the adaptation of golden Camellia to karst habitats (**Figure 4C**).

### 3.6 DEGs of root gene expression among different golden Camellia

**Figure 5:**
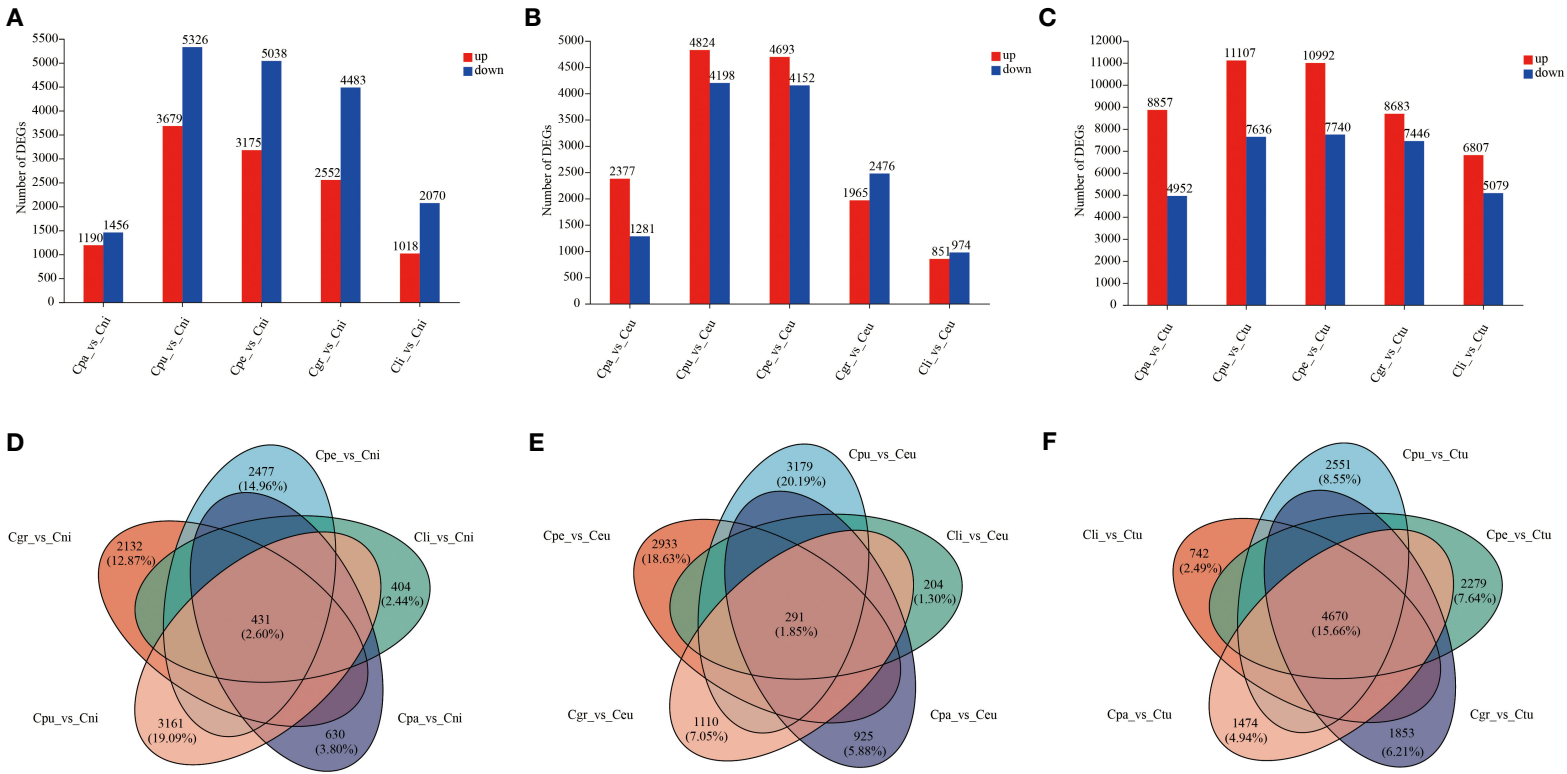
DEGs of root gene expression among different golden Camellia. **(A)** The bar graph of DEGs at Cpa_vs_Cni, Cpu_vs_Cni, Cpe_vs_Cni, Cgr_vs_Cni, and Cli_vs_Cni. **(B)** The bar graph of DEGs at Cpa_vs_Ceu, Cpu_vs_Ceu, Cpe_vs_Ceu, Cgr_vs_Ceu, and Cli_vs_Ceu. **(C)** The bar graph of DEGs at Cpa_vs_Ctu, Cpu_vs_Ctu, Cpe_vs_Ctu, Cgr_vs_Ctu, and Cli_vs_Ctu. **(D)** The Venn diagram of DEGs at Cpa_vs_Cni, Cpu_vs_Cni, Cpe_vs_Cni, Cgr_vs_Cni, and Cli_vs_Cni. **(E)** The Venn diagram of DEGs at Cpa_vs_Ceu, Cpu_vs_Ceu, Cpe_vs_Ceu, Cgr_vs_Ceu, and Cli_vs_Ceu. **(F)** The Venn diagram of DEGs at Cpa_vs_Ctu, Cpu_vs_Ctu, Cpe_vs_Ctu, Cgr_vs_Ctu, and Cli_vs_Ctu.

Cni, Ceu, and Ctu were selected and compared with golden Camellia (Cpu, Cpe, Cgr, Cli, and Cpa), respectively, to investigate the differences in the adaptation of different golden Camellia to karst habitats (**Figure 5**). In the comparisons of DEGs in Cpa_vs_Cni, Cpu_vs_Cni, Cpe_vs_Cni, Cgr_vs_Cni, Cli_vs_Cni, Cpa_vs_Ceu, Cpu_vs_Ceu, and Cpe_vs_Ceu, the numbers of downregulated genes were higher than the numbers of upregulated genes in each comparison group (**Figures 5A, B**; **Table S1**).

However, in Cpa_vs_Ctu, Cpu_vs_Ctu, Cpe_vs_Ctu, Cgr_vs_Ctu, Cli_vs_Ctu, Cgr_vs_Ceu, and Cli_vs_Ceu, the upregulated genes of DEGs were higher than the downregulated genes (**Figures 5B, C**). Moreover, the Cpu-related group had the most DEGs among all comparison groups (Cpu_vs_Cni; Cpu_vs_Ceu; Cpu_vs_Ctu), followed by the Cpe-related (Cpe_vs_Cni; Cpe_vs_Ceu; Cpe_vs_Ctu) group, while the Cpa-related group (Cpa_vs_Cni; Cpa_vs_Ceu; Cpa_vs_Ctu) and Cli-related group (Cli_vs_Cni; Cli_vs_Ceu; Cli_vs_Ctu) had lower DEGs (**Figure 5**). This indicated that there were differences in the ability of different golden Camellia growing in karst soil to adapt to karst habitats. In addition, there were only 2.60% (431) and 1.85% (291) of common DEGs in the Cni-related group (Cpa_vs_Cni, Cpu_vs_Cni, Cpe_vs_Cni, Cgr_vs_Cni, and Cli_vs_Cni) and the Ceu-related group (Cpa_vs_Ceu, Cpu_vs_Ceu, Cpe_vs_Ceu, Cgr_vs_Ceu, and Cli_vs_Ceu), while there were 15.66% (4,670) of common DEGs in the Ctu-related group (Cpa_vs_Ctu, Cpu_vs_Ctu, Cpe_vs_Ctu, Cgr_vs_Ctu, and Cli_vs_Ctu) (**Figures 5D–F**).

### 3.7 The annotation and functional enrichment of DEGs in different groups

**Figure 6:**
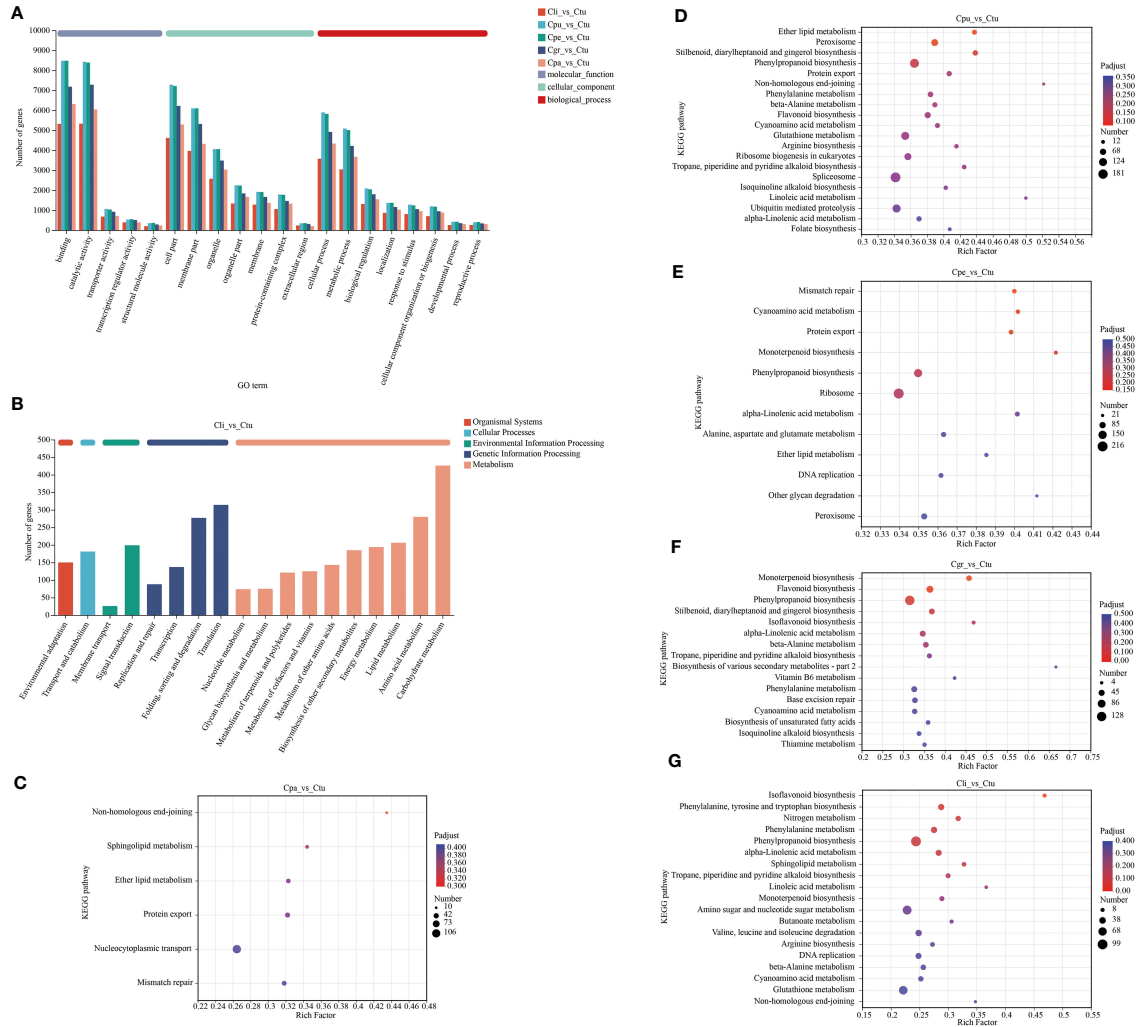
Functional annotation and enrichment of DEGs in the root of golden Camellia. **(A)** Functional annotation of DEGs for Cpa_vs_Ctu, Cpu_vs_Ctu, Cpe_vs_Ctu, Cgr_vs_Ctu, and Cli_vs_Ctu based on the GO database. **(B)** Functional annotation of DEGs for Cli_vs_Ctu based on the KEGG database. **(C–G)** Functional enrichment of DEGs based on the KEGG database. Cpa_vs_Ctu **(C)**. Cpu_vs_Ctu **(D)**. Cpe_vs_Ctu **(E)**. Cgr_vs_Ctu **(F)**. Cli_vs_Ctu **(G)**. *p*_adjust_ < 0.5.

The annotation and functional enrichment of DEGs were performed by the GO and KEGG database to further analyze the regulation pathways of golden Camellia in different habitats. Based on the above DEGs, the largest number of DEGs in the Ctu-related group was selected for annotation and functional enrichment in this study (**Figures 6**, **S5**).

In GO and KEGG database annotation, the distribution trend of DEGs in each Ctu-related comparison group was basically the same in each GO term and KEGG functional pathway (**Figures 6A, B**, **S5**). Binding, catalytic activity, cell part, membrane part, and cellular process were the five GO terms with the number of DEGs in the annotation in each Ctu-related comparison group. Carbohydrate metabolism, amino acid metabolism, folding, sorting, and degradation were the main functional pathways in KEGG database annotation in each Ctu-related comparison group, which suggests the commonality of the regulatory pathways of Cpa, Cpu, Cpe, Cgr, and Ctu in habitats.

The functional enrichment in each Ctu-related comparison group was shown to further analyze the link between DEGs and functions based on KEGG pathway level 3 (**Figures 6C–G**). In Cpa_vs_Ctu, non-homologous end-joining was the most enriched (rich factor = 0.43), while nucleocytoplasmic transport was the most enriched (number = 106) (**Figure 6C**). Also, non-homologous end-joining was also the most enriched in Cpu_vs_Ctu (rich factor = 0.52) (**Figure 6D**). Among DEGs in Cpu_vs_Ctu, they were enriched in phenylpropanoid biosynthesis, stilbenoid, diarylheptanoid and gingerol biosynthesis, and flavonoid biosynthesis way related to secondary metabolites, etc. Pathways related to secondary metabolites were enriched not only in DEGs of Cpu_vs_Ctu, but also in those of Cpe_vs_Ctu, Cgr_vs_Ctu, and Cli_vs_Ctu, especially in phenylpropanoid biosynthesis (number: Cpu_vs_Ctu = 148; Cpe_vs_Ctu = 142; Cgr_vs_Ctu = 128; Cli_vs_Ctu = 99) (**Figures 6D–G**). These results suggested that the adaptation of golden Camellia growing in karst soil is one of the mechanisms by regulating its own metabolic substances.

### 3.8 The secondary metabolic biosynthetic pathways in different golden Camellia species growing in karst soil

**Figure 7:**
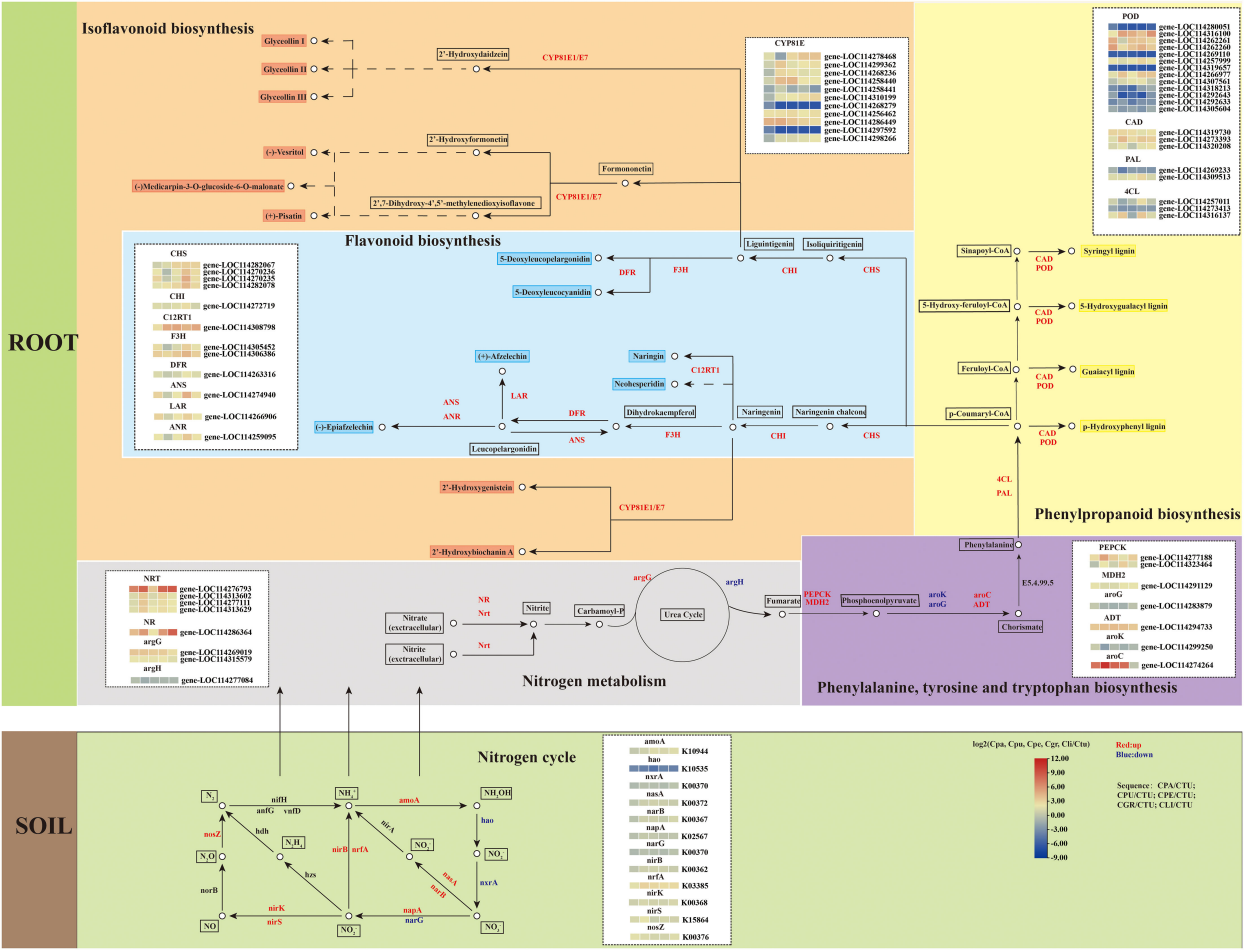
Gene expression of golden Camellia growing in karst soil in the secondary metabolite synthesis pathways.

After annotation and functional enrichment of the DEGs in the KEGG database, the results showed that the DEGs were significantly enriched in secondary metabolic biosynthetic pathways. Gene expression profiles were shown in the pathway of secondary metabolite synthesis to illustrate the adaptation of golden Camellia to karst habitats, which was done using Ctu as the control (**Figure 7**).

The DEGs in the Ctu-related comparison groups were mainly upregulated in flavonoid biosynthesis, phenylpropanoid biosynthesis, isoflavonoid biosynthesis, and nitrogen metabolism. In the isoflavonoid biosynthesis, there were 11 DEGs related to *CYP81E* enriched, namely, 8 upregulated genes and 3 downregulated genes. In flavonoid biosynthesis, the upregulated genes include *CHS* and *CHI*. In addition, the upregulation of genes involved in flavonoid biosynthesis accelerated the production of various flavonoid species, such as *C12RT1*, which was upregulated on Cpa, Cpu, Cpe, Cgr, and Cli. Compared to Ctu, the expressions of *CAD* and *POD* in Cpa, Cpu, Cpe, and Cli were increased. In soil rhizosphere microorganisms, there were also related DEGs in the process of nitrogen cycle, and the upregulated genes were more than the downregulated genes, such as *amoA* and *nirB*. This suggests that rhizosphere microorganism may assist the adaptation of golden Camellia to karst habitats by accelerating nitrogen cycling.

## 4 Discussion

By combining metagenome and transcriptome approaches, we analyzed the differences in rhizosphere microbial structure and gene expression in roots of golden Camellias in order to understand the adaptation mechanisms of golden Camellia in karst environments.

**Figure 8:**
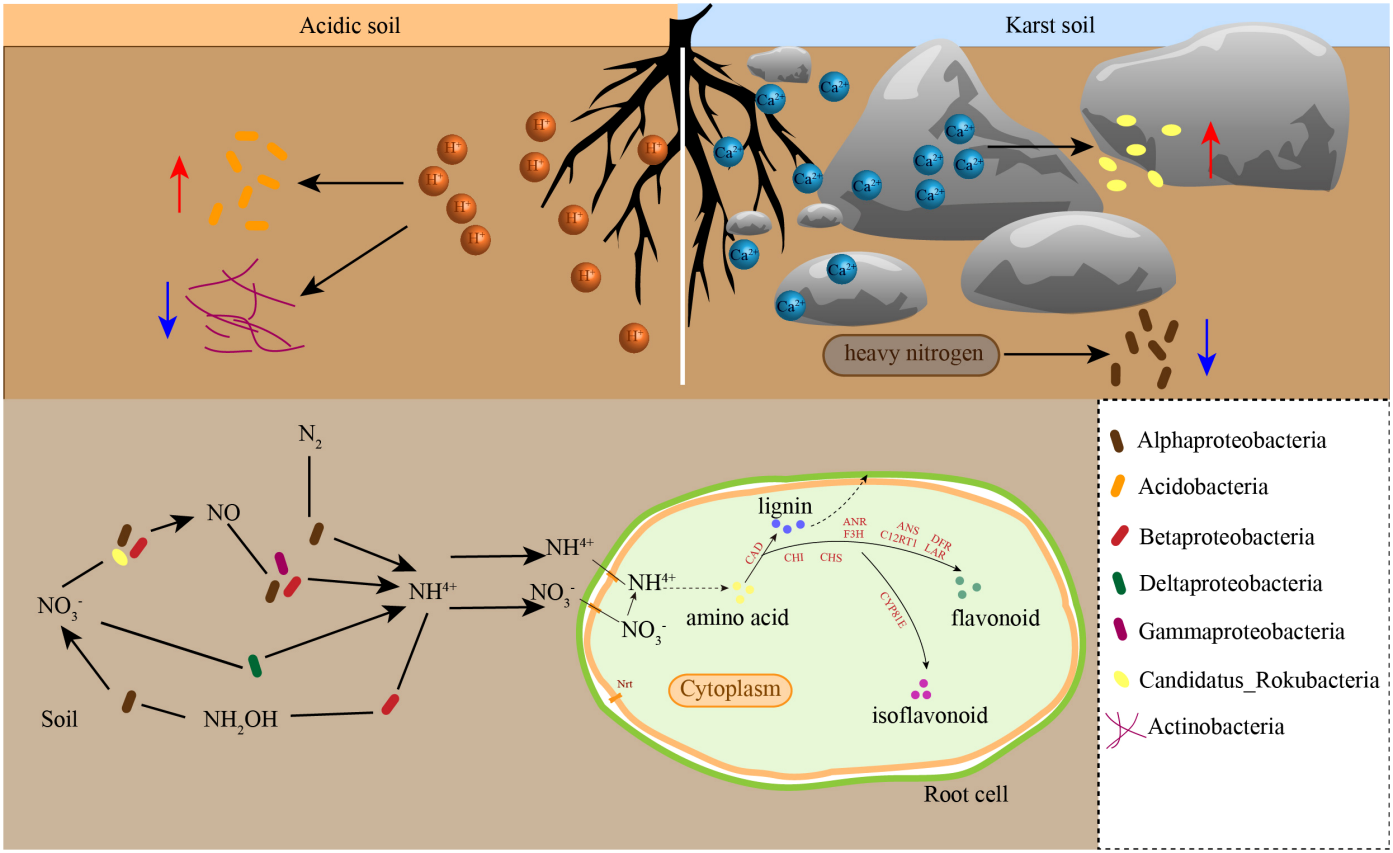
Model of effects of soil properties on rhizosphere microbial communities and their role in nitrogen cycling. The blue arrow indicates the benefit of bacterial growth, and the red arrow represents the inhibition of bacterial growth.

First of all, in terms of rhizosphere microbial composition, Cni, Ceu, and Ctu growing in acidic soil have high similarity, while Cpa has more similarity with Cpu, Cpe, Cgr, and Cli, which grow in karst soil. In addition, Cpa is the more drought-resistant golden Camellia growing in acidic soil (Su and Mo, 1988). In terms of rhizosphere soil microbial composition, the proportion of Proteobacteria at the phylum level was the highest among the eight species of golden Camellia. Proteobacteria is the largest bacterial phylum, including Alphaproteobacteria, Betaproteobacteria, Deltaproteobacteria, and Gammaproteobacteria, which can survive under stress conditions such as drought, heavy metals, and acidic environment (Wang et al., 2019; Liu et al., 2021). The results of this study also found that Proteobacteria, especially Alphaproteobacteria, had the highest functional contribution in the top seven abundance in rhizosphere soil (**Figure 3J**). Alphaproteobacteria include nitrogen-fixing and nitrifying bacteria that reduce ammonia and ammonium to nitrate, while Betaproteobacteria reduce nitrate to nitrite (Einsle, 2011; Li et al., 2012). Ceu had the highest proportion of Alphaproteobacteria, followed by Ctu, and genes related to NH_4_^+^ reduction to NO_3_^−^, such as *hao* and *nxrA*, also had higher gene abundance in the Ctu-related comparison groups (**Figures 2F**, **7**), while the proportion of Betaproteobacteria was higher in Cpa and Cpu. The abundance of Alphaproteobacteria had been reported to be inversely proportional to high nitrogen concentration (Li et al., 2019; Zhu et al., 2022a). Moreover, at the class level, Deltaproteobacteria and Betaproteobacteria were more abundant in karst soil than in acidic soil. Acidobacteria belonged to acidophilus, and its proportion in Cni, Ceu, and Ctu was significantly higher than that in golden Camellia growing in karst soil. As an important component of microbial community, it has important ecological functions by promoting carbon, nitrogen, and sulfur metabolism in soil (Kalam et al., 2020). Different from Acidobacteria, the proportion of Candidatus_Rokubacteria in Cpa, Cpu, Cpe, Cgr, and Cli was significantly higher than that of Cni, Ceu, and Ctu. Candidatus_Rokubacteria growth and reproduction are promoted by soil nitrogen, calcium, magnesium, and other elements (Ivanova et al., 2021; Zhu et al., 2022a). Among the functions of rhizosphere soil microorganisms in karst habitats, ABC transporters and quorum sensing have scores in high linear discriminant analysis, and the two most important contributors to these functions were Alphaproteobacteria and Candidatus_Rokubacteria (**Figure 3**). These results indicated that the karst habitats affected the survival of Candidatus_Rokubacteria and Candidatus_Rokubacteria significantly affected the function of soil microbial community in karst habitats. Actinobacteria was the third most abundant phylum at the phylum level, with higher proportions in Cpu, Cpe, and Cgr (**Figure 2E**). Actinobacteria have the ability to decompose organic matter, especially cellulose and chitin (Liu et al., 2014; Liu et al., 2016; Li et al., 2019; Zhu et al., 2022a). In addition, the decomposition of nitride-containing substances can increase the nitrogen content in soil, which may enhance the absorption of carbon and nitrogen by plants, and the absorption of nitrogen in plants can promote the production of secondary metabolites, thereby alleviating the damage caused by external stress (**Figure 8**).

Soil properties affect the composition of rhizosphere soil microorganisms in different habitats, which, in turn, affect their functional pathways and may assist in the adaptation of golden Camellia to karst habitats. The results showed that ABC transporters and quorum sensing were most different in rhizosphere soil microorganisms of golden Camellia growing in karst soil compared with those growing in acidic soil. As one of the largest protein families, ABC transporters are responsible for transporting carbohydrates, lipids, and proteins (Qu et al., 2020). Significantly, ABC transporters in rhizosphere microorganisms can mediate the exchange of substances between the roots and soil, which may be beneficial to the absorption of ferrite, phosphorus, and zinc ion by the roots of plants, thus promoting its growth and development (Ryan et al., 2001). Quorum sensing is due to a certain density of microorganisms, which allows bacteria to communicate with each other (Basavaraju et al., 2016). CAZy genes include glycosyl transferases (GT), glycoside hydrolases (GH), carbohydrate esterases (CE), auxiliary activities (AA), polysaccharide lyases (PL), and carbohydrate-binding modules (CBM), which have the functions of degradation and modification. GT and GH accounted for the highest proportion in the rhizosphere soil microorganisms of the eight species of golden Camellia. GT is responsible for the synthesis of sugars and sugar-containing substances (Lairson et al., 2008). GH has the ability to decompose organic matter to form various monosaccharides and oligosaccharides, which provide energy for microorganisms themselves and plants (Manoharan et al., 2015).

When plants are exposed to abiotic stress, their gene expression levels will also change in response to external stress. Drought and high calcium stress in karst regions have a certain negative effect on the growth of plants, which are not adaptive to karst regions. Calcium is an essential mineral element for plant growth and development. It plays an important role in cell components, signal transduction, and other aspects (Yang et al., 2008). In addition, when plants are exposed to high calcium environment for a long time, the synthesis of various species, including organic acids and osmotic substances, helps plants adapt to the high calcium environment (Zhang et al., 2021). Studies have shown that the secondary metabolic contents of flavonoids, anthocyanins, saponins in apple, and *Panax notoginseng* are increased in a high-calcium environment (Zu et al., 2022). High concentration of calcium in the soil will cause an osmotic toxic effect on the growth of golden Camellia, reducing the activities of various antioxidant enzymes, such as superoxide dismutase (SOD), catalase (CAT), and peroxidase (POD), causing serious oxidative damage to plants (Yang and Fang, 2022). In the present study, KEGG enrichment analysis of DEGs in the Ctu-related comparison groups, especially in Cpu_vs_Ctu, Cpe_vs_Ctu, Cgr_vs_Ctu, and Cli_vs_Ctu, was found to be enriched in multiple secondary metabolic synthesis pathways, including phenylpropanoid biosynthesis, flavonoid biosynthesis, and isoflavonoid biosynthesis. Phenylpropanoid, flavonoid, and isoflavonoid can eliminate free radicals in plants and enhance their antioxidant capacity to reduce oxidative damage caused by high calcium environment (Li et al., 2018). Moreover, some flavonoids, such as anthocyanins, can enhance the color of plant leaves and maintain a high photosynthetic capacity of plants, which is an important adaptive mechanism for golden Camellia to adapt to the high-calcium environment (Liu et al., 2022a). Firstly, in isoflavonoid biosynthesis, eight genes were found to be upregulated and related to *CYP81E*, which is an important metabolic enzyme in various isoflavones’ synthesis in roots of Cpu, Cpe, Cgr, and Cli compared with Ctu. The expression of *CYP81E*-related genes was mainly related to the accumulation of glyceollin I/II/III, 2′-Hydroxygenistein, 2′-Hydroxybiochanin A, vesritol, medicarpin-3-O-glucoside-6-O-malonate, and pisatin (Liu et al., 2003; Liu et al., 2022b; Wang et al., 2023). Likely, during soybean germination, glyceollin I accumulation can eliminate reactive oxygen species (ROS) caused by microbial stress and alleviate membrane lipid peroxidation (Jeon et al., 2012). The synthesis of pisatin can enhance the resistance of pea seeds to osmotic stress (Brosowska-Arendt et al., 2014). The expression of *CHS* in flavonoid biosynthesis pathway was downregulated in Feizixiao lychee fruit after high calcium treatment which may cause oxidative damage (Shui et al., 2022). Therefore, the upregulated expression of *CHS* in the roots of golden Camellia growing in karst soil may help its adaptation to the high Ca^2+^ environment. The synthesis of a variety of flavonoids may promote the adaptation of golden Camellia to karst habitats. Flavonoids and isoflavones have similar effects on plants and can mitigate oxidative damage caused by scavenging ROS produced when plants are stressed (Hernandez et al., 2009). In drought-tolerant rice cultivars, oxidative damage induced by drought stress is alleviated by the accumulation of flavonoids, including naringin (Dey and Bhattacharjee, 2020). In the roots of salt-tolerant wheat varieties, genes related to phenylpropanoid biosynthesis, including *4CL* and *CAD*, were significantly upregulated (Yue et al., 2022). In the phenylpropanoid biosynthesis pathway, the upregulated expression of *CAD* and *POD* involved the production of various lignin. Lignin can provide enhanced cell support that improves plant body support and water removal, thereby increasing plant drought resistance (Cabane et al., 2012). A complex interplay exists between the nitrogen metabolic pathway and secondary metabolite synthesis. The synthesis of secondary metabolites such as flavonoids, isoflavones, and phenylpropanoids derived from phenylalanine and tyrosine is also affected by the uptake of carbon and nitrogen, so there is a complex interaction between the nitrogen metabolic pathway and the synthesis of secondary metabolites (Garcia-Calderon et al., 2020). The genes related to *NRT* and *NR* can enhance the uptake of extracellular nitrate and nitrite, with nitrogen cycling in the rhizosphere soil facilitated by microorganisms (Zhao et al., 2018). In the present study, the roots of golden Camellia growing in karst areas demonstrated the upregulation of genes related to *NRT* and *NR*. Golden Camellia’s adaptation to karst regions may associate with the rhizosphere microbe composition and the expression of genes in root, especially the genes that were involved in secondary metabolite synthesis pathways.

## 5 Conclusion

In this study, metagenomic and transcriptome sequencing were used to analyze the differences in rhizosphere soil microbial composition and root gene expression between golden Camellia growing in acidic soil and that growing in karst soil to compare and investigate their differences in adaptation to karst areas. Compared to the expression level of root genes in golden Camellia growing in acidic soil, those in karst soil are upregulated for genes in the secondary metabolic synthesis pathway and for genes involved in nitrogen transport. Thus, through the absorption of some nitrogen substances to adapt to the karst habitat, golden Camellia may enhance secondary metabolite synthesis. The abundance of Candidatus_Rokubacteria in rhizosphere soil microorganisms of golden Camellia growing in karst soil is significantly higher than that in acidic soil, which makes them have stronger ABC transporters and quorum sensing functions that may affect the transport of materials. Soil microorganisms in the rhizosphere of golden Camellia grown in karst soil exhibit a significant capability to improve soil nitrogen cycling. Rhizosphere microorganisms have the potential to enhance nitrogen absorption in the roots of golden Camellia by improving material transport in the soil. Consequently, this assists golden Camellia in synthesizing secondary metabolites, which could significantly contribute to the survival and growth of golden Camellia in karst regions.

## Data availability statement

The original contributions presented in the study are publicly available. This data can be found here: https://www.ncbi.nlm.nih.gov/sra/PRJNA928560.

## Author contributions

SC, YL, and XW designed the study; JL, HJ, LZ and YH performed the experiment; QX, LZ and QY analyzed the data and drafted the manuscript; HJ, YL, and SC also collected the samples. All authors contributed to the article and approved the submitted version.

## Supplementary material

Supplementary Figure 1 Picture of sampling of golden Camellia growing in acidic soil and karst soil. **(A)** Sampling of Cni. **(B)** Sampling of Cli.

Supplementary Figure 2 Expression level of each root sample. The quantitative index was TPM.

Supplementary Figure 3 Differences in rhizosphere microbial community composition. **(A)** Between Cni, Ceu, Ctu, and Cpa at the phylum level. **(B)** Between Cni, Ceu, Ctu, and Cpa at the class level. **(C)** Between Cpu, Cpe, Cgr, and Cli at the phylum level. **(D)** Between Cpu, Cpe, Cgr, and Cli at the phylum level and class level.

Supplementary Figure 4 Gene functions of rhizosphere microbial communities. **(A)** LEfSe analysis at KEGG pathway level 3 with significant differences in Cni and Cpu. **(B)** In Cni and Cpe. **(C)** In Cni and Cgr.

Supplementary Figure 5 Functional annotation of DEGs based on the KEGG database. **(A)** In Cpa_vs_Ctu. **(B)** In Cpu_vs_Ctu. **(C)** In Cpe_vs_Ctu. **(D)** In Cgr_vs_Ctu.

## References

[B1] Basavaraju M. Sisnity V. Palaparthy R. Addanki P. (2016). Quorum quenching: Signal jamming in dental plaque biofilms. J. Dent. Sci. 11, 349–352. doi: 10.1016/j.jds.2016.02.002 30894996 PMC6395279

[B2] Brosowska-Arendt W. Gallardo K. Sommerer N. Weidner S. (2014). Changes in the proteome of pea (Pisum sativum L.) seeds germinating under optimal and osmotic stress conditions and subjected to post-stress recovery. Acta Physiologiae Plantarum 36, 795–807. doi: 10.1007/s11738-013-1458-8

[B3] Cabane M. Afif D. Hawkins S. (2012). “Chapter 7 - Lignins and abiotic stresses,” in Advances in Botanical Research. Eds. Jouann L. Lapierre C. (San Diego, CA, USA: Academic Press) 61, 219–262. doi: 10.1016/B978-0-12-416023-1.00007-0

[B4] Chai S. Fu R. Zou R. (2021). Effects of different calcium ion concentrations onphotosynthetic and physiological indexes of calcicole-typeand calcifuge-type golden Camellia. Guihaia 41, 167–176. doi: 10.11931/guihaia.gxzw201911017

[B5] Deng Y. Jiang Z. Cao J. Lan F. N. (2004). Characteristics com- parison of the leaf anatomy of Cyclobalanopsis glauca and its adaption to the environment of typical karst peak cluster areas in Nongla. Guihaia 24 (4), 317–322. doi: 1000-3142(2004)04-0317-06.

[B6] Dey N. Bhattacharjee S. (2020). Accumulation of polyphenolic compounds and osmolytes under dehydration stress and their implication in redox regulation in four indigenous aromatic rice cultivars. Rice Sci. 27, 329–344. doi: 10.1016/j.rsci.2020.05.008

[B7] Dolman N. J. Tepikin A. V. Petersen O. H. (2003). Generation and modulation of cytosolic Ca2+ signals in pancreatic acinar cells: techniques and mechanisms. Biochem. Soc. Trans. 31, 947–949. doi: 10.1042/bst0310947 14505455

[B8] Einsle O. (2011). Structure and function of formate-dependent cytochrome c nitrite reductase, NrfA. Meth. Enzymol. 496, 399–422. doi: 10.1016/B978-0-12-386489-5.00016-6 21514473

[B9] Garcia-Calderon M. Perez-Delgado C. M. Palove-Balang P. Betti M. Marquez A. J. (2020). Flavonoids and isoflavonoids biosynthesis in the model legume lotus japonicus; connections to nitrogen metabolism and photorespiration. Plants (Basel, Switzerland) 9 (6), 774. doi: 10.3390/plants9060774 32575698 PMC7357106

[B10] Guo Y. Yu H. Yang M. Kong D. Zhang Y. (2018). Effect of drought stress on lipid peroxidation, osmotic adjustment and antioxidant enzyme activity of leaves and roots of lycium ruthenicum murr. Seedling. Russ. J. Plant Physiol. 65, 244–250. doi: 10.1134/S1021443718020127

[B11] Hernandez I. Alegre L. Van Breusegem F. Munne-Bosch S. (2009). How relevant are flavonoids as antioxidants in plants? Trends Plant Sci. 14, 125–132. doi: 10.1016/j.tplants.2008.12.003 19230744

[B12] Ivanova A. A. Oshkin I. Y. Danilova O. V. Philippov D. A. Ravin N. Dedysh S. N. (2021). Rokubacteria in Northern peatlands: habitat preferences and diversity patterns. Microorganisms 10 (1), 11. doi: 10.3390/microorganisms10010011 35056460 PMC8780371

[B13] Jarrett H. W. Brown C. J. Black C. C. Cormier M. J. (1982). Evidence that calmodulin is in the chloroplast of peas and serves a regulatory role in photosynthesis. J. Biol. Chem. 257, 13795–13804. doi: 10.1016/S0021-9258(18)33519-1 6292207

[B14] Jeon H. Seo D. Shin H. Lee S. (2012). Effect of aspergillus oryzae-challenged germination on soybean isoflavone content and antioxidant activity. J. Agric. Food Chem. 60, 2807–2814. doi: 10.1021/jf204708n 22409158

[B15] Jin W. Long Y. Fu C. Zhang L. Xiang J. Wang B.. (2018). Ca2+ imaging and gene expression profiling of Lonicera Confusa in response to calcium-rich environment. Sci. Rep. 8 (1), 7068. doi: 10.1038/s41598-018-25611-5 29728644 PMC5935734

[B16] Kalam S. Basu A. Ahmad I. Sayyed R. El-Enshasy H. Dailin D.. (2020). Recent understanding of soil acidobacteria and their ecological significance: A critical review. Front. Microbiol. 11, 580024. doi: 10.3389/fmicb.2020.580024 33193209 PMC7661733

[B17] Lairson L. L. Henrissat B. Davies G. J. Withers S. G. (2008). Glycosyltransferases: structures, functions, and mechanisms. Annu. Rev. Biochem. 77, 521–555. doi: 10.1146/annurev.biochem.76.061005.092322 18518825

[B18] Li X. He Y. Xie C. Zu Y. Zhan F. Mei X.. (2018). Effects of UV-B radiation on the infectivity of Magnaporthe oryzae and rice disease-resistant physiology in Yuanyang terraces. Photochemical & photobiological sciences: Official journal of the European Photochemistry Association and the European Society for Photobiology 17 (1), 364–364. doi: 10.1039/c8pp90006j 29110008

[B19] Li J. Lin W. Luo J. Tian G. (2012). Community structure of microbes involved in nitrification and denitrification in typical shrimp-farming water. Weishengwu Xuebao 52, 478–488. 22799213

[B20] Li Y. Pan F. Yao H. (2019). Response of symbiotic and asymbiotic nitrogen-fixing microorganisms to nitrogen fertilizer application. J. Soils Sediments 19, 1948–1958. doi: 10.1007/s11368-018-2192-z

[B21] Li W. Xu F. Chen S. Zhang Z. Zhao Y. Jin Y.. (2014). A comparative study on Ca content and distribution in two Gesneriaceae species reveals distinctive mechanisms to cope with high rhizospheric soluble calcium. Front. Plant Sci. 5, 647. doi: 10.3389/fpls.2014.00647 25477893 PMC4238373

[B22] Liu J. Ahmad N. Hong Y. Zhu M. Zaman S. Wang N.. (2022b). Molecular characterization of an isoflavone 2 ‘-hydroxylase gene revealed positive insights into flavonoid accumulation and abiotic stress tolerance in safflower. Molecules (Basel, Switzerland) 27 (22), 8001. doi: 10.3390/molecules27228001 36432102 PMC9697648

[B23] Liu C. Huang Y. Liang Y. (2022a). Adaptive mechanism exploration of camellia limonia in karst high calcium environment by integrative analysis of metabolomics and metagenomics. Trop. Plant Biol. 15, 22–39. doi: 10.1007/s12042-021-09308-0

[B24] Liu Y. Huang Y.-m. Zeng Q.-c. (2016). Soil bacterial communities under different vegetation types in the loess plateau. Huanjing Kexue 37, 3931–3938. doi: 10.13227/j.hjkx.2016.10.035 29964429

[B25] Liu C. Huhman D. Sumner L. Dixon R. (2003). Regiospecific hydroxylation of isoflavones by cytochrome P450 81E enzymes from Medicago truncatula. Plant J. 36, 471–484. doi: 10.1046/j.1365-313X.2003.01893.x 14617078

[B26] Liu J. Sui Y. Yu Z. Shi Y. Chu H. Jin J.. (2014). High throughput sequencing analysis of biogeographical distribution of bacterial communities in the black soils of northeast China. Soil Biol. Biochem. 70, 113–122. doi: 10.1016/j.soilbio.2013.12.014

[B27] Liu B. Yao J. Ma B. Chen Z. Zhao C. Zhu X.. (2021). Microbial community profiles in soils adjacent to mining and smelting areas: Contrasting potentially toxic metals and co-occurrence patterns. Chemosphere 282, 130992. doi: 10.1016/j.chemosphere.2021.130992 34087556

[B28] Manoharan L. Kushwaha S. Hedlund K. Ahren D. (2015). Captured metagenomics: large-scale targeting of genes based on “sequence capture” reveals functional diversity in soils. DNA Res. 22, 451–460. doi: 10.1093/dnares/dsv026 26490729 PMC4675713

[B29] Qu J. Chen T. Yao M. Wang Y. Xiao W. Li B. (2020). [ABC transporter and its application in synthetic biology]. Sheng wu gong cheng xue bao = Chin. J. Biotechnol. 36, 1754–1766. doi: 10.1093/dnares/dsv026 33164454

[B30] Rong L. Wang S. Liu N. Yang L. (2005). Leaf anatomical characters and its ecological adaptation of the pioneer species in the karst mountain area-with a special reference to the Huajiang Canyon of Guizhou. J. Mount 23, 35–42. doi: 10.16089/j.cnki.1008-2786.2005.01.004

[B31] Ryan P. Delhaize E. Jones D. (2001). Function and mechanism of organic anion exudation from plant roots. Annu. Rev. Plant Physiol. Plant Mol. Biol. 52, 527–560. doi: 10.1146/annurev.arplant.52.1.527 11337408

[B32] Shui X. Wang W. Ma W. Yang C. Zhou K. (2022). Mechanism by which high foliar calcium contents inhibit sugar accumulation in feizixiao lychee pulp. Horticulturae 8 (11), 1044. doi: 10.3390/horticulturae8111044

[B33] Su Z. Mo X. (1988). Geographical distribution of camellia section chrysantha from China. Guihaia 8, 77–83.

[B34] Wang X. Gao P. Li D. Liu J. Yang N. Gu W.. (2019). Risk assessment for and microbial community changes in Farmland soil contaminated with heavy metals and metalloids. Ecotoxicol. Environ. Saf. 185, 109685. doi: 10.1016/j.ecoenv.2019.109685 31541947

[B35] Wang Y. Li Z. Ahmad N. Sheng X. Iqbal B. Naeem M.. (2023). Unraveling the functional characterization of a jasmonate-induced flavonoid biosynthetic CYP45082G24 gene in Carthamus tinctorius. Funct. Integr. Genomics 23 (2), 172. doi: 10.1007/s10142-023-01110-3 37212893

[B36] Wang C.-Y. Wang S.-J. Rong L. Luo X.-Q. (2011). Analyzing about characteristics of calcium content and mechanisms of high calcium adaptation of common pteridophyte in Maolan karst area of China. Chin. J. Plant Ecol. 35, 1061–1069. doi: 10.3724/SP.J.1258.2011.01061

[B37] Wei S. Lu Y. Ye Q. Tang S. (2017). Population genetic structure and phylogeography of camellia flavida (Theaceae) based on chloroplast and nuclear DNA sequences. Front. Plant Sci. 8, 718. doi: 10.3389/fpls.2017.00718 28579991 PMC5437371

[B38] Yang hy Fang W. (2022). Effects of different calcium supply levels on the growth, mineral element absorption, and related physiological and biochemical characteristics of Roxobin seedlings. J. Fruit Trees 39, 1891–1902.

[B39] Yang Q. Wei X. Zeng X. Ye W. Yin X. Zhang-Ming W.. (2008). Seed biology and germination ecophysiology of Camellia nitidissima. For. Ecol. Manag. 255, 113–118. doi: 10.1016/j.foreco.2007.08.028

[B40] Yue Z. Chen Y. Wang Y. Zheng L. Zhang Q. Liu Y.. (2022). Halotolerant Bacillus altitudinis WR10 improves salt tolerance in wheat via a multi-level mechanism. Front. Plant Sci. 941388, 13. doi: 10.3389/fpls.2022.941388 PMC9330482 35909740

[B41] Zhang X. Liu L. Gong J. Tang J. Tang M. Yi Y. (2017). Comparison of calcium distributions leaf cells of Carpinus pubescens and camellia oleifera under drought and calcium stress. Pak. J. Bot. 49, 2139–2143.

[B42] Zhang Y. Liu Y. Wang Y. Yang B. (2021). Characteristics of organic acid secretion by roots of typical ferns selaginella tamariscina in karst area. J. North-East Forestry Univ. 49 (4), 52–55, 61. doi: 10.13759/j.cnki.dlxb.2021.04.009

[B43] Zhao L. Zhang W. Yang Y. Li Z. Li N. Qi S.. (2018). The Arabidopsis NLP7 gene regulates nitrate signaling via NRT1.1-dependent pathway in the presence of ammonium. Sci. Rep. 8 (1), 1–13. doi: 10.1038/s41598-018-20038-4 29367694 PMC5784019

[B44] Zhu L. Qin D. Zhao L. (2021). Leaf epidermal micromorphological features and their systematicSignificance of six wild species of camellia chrysantha. Bull. Botanical Res. 41, 841–850. doi: 10.7525/j.issn.1673-5102.2021.06.001

[B45] Zhu X. Tang J. Qin H. Bai K. Chen Z. Zou R.. (2022a). Contrasting adaptation mechanisms of golden camellia species to different soil habitats revealed by nutrient characteristics. Agronomy 12 (7), 1511. doi: 10.3390/agronomy12071511

[B46] Zhu X. Tang J. Tao Y. (2022b). Difference in calcium speciation of leaves of golden Camellia species from calcareous soil and acidic soil habitats. Guihaia 42 (3), 442–451. doi: 10.11931/guihaia.gxzw202203052

[B47] Zu Y. Mei X. Li B. Li T. Li Q. Qin L.. (2022). Effects of calcium application on the yields of flavonoids and saponins inPanax notoginsengunder cadmium stress. J. Environ. Anal. Chem. 102, 4208–4219. doi: 10.1080/03067319.2020.1781835

