# Supplementary material for: Combined analysis of metagenome and transcriptome revealed the adaptive mechanism of different golden Camellia species in karst regions
Source: Front Plant Sci. 2023 Nov 20;14:1180472. doi: 10.3389/fpls.2023.1180472 (PMC10699447; doi:10.3389/fpls.2023.1180472)
Supplement: Supplementary file 1 [file Table_1.docx]

| Table S1 Soil characteristics of eight species golden Camellia | | |
| --- | --- | --- |
| Species | Soil pH | Soil Ca content (g kg^-1^) |
| *Camellia nitidissima*  CNI | 3.81±0.23d | 0.40±0.16c |
| *Camellia euphlebia*  CEU | 5.21±0.57bc | 0.48±0.10c |
| *Camellia tunghinensis*  CTU | 5.00±0.37c | 0.60±0.12c |
| *Camellia parvipetala*  CPA | 5.86±0.15b | 1.47±0.33c |
| *Camellia pubipetala*  CPU | 7.28±0.13a | 5.96±1.49a |
| *Camellia perpetua*  CPE | 7.00±0.51a | 3.31±0.96b |
| *Camellia grandis*  CGR | 6.98±0.59a | 5.88±1.62a |
| *Camellia limonia*  CLI | 6.93±0.17a | 3.49±0.57b |
