# Supplementary material for: Combined analysis of metagenome and transcriptome revealed the adaptive mechanism of different golden Camellia species in karst regions
Source: Front Plant Sci. 2023 Nov 20;14:1180472. doi: 10.3389/fpls.2023.1180472 (PMC10699447; doi:10.3389/fpls.2023.1180472)
Supplement: Supplementary file 2 [file Table_2.docx]

Table S2 Characteristics of DNA-sequencing results of different soil samples

| Samples | Raw reads | Clean reads | Clean read percent in raw reads(%) | Contigs | N50(bp) | N90(bp) | ORFs | Total Length(bp)of ORFs | Average Length(bp) of ORFs | Max(bp)of ORFs | Min(bp)of ORFs |
| --- | --- | --- | --- | --- | --- | --- | --- | --- | --- | --- | --- |
| CNI_S1 | 49365436 | 48664304 | 98.58 | 567370 | 590 | 343 | 679479 | 281995494 | 415.02 | 7446 | 102 |
| CNI_S2 | 50919038 | 50198386 | 98.58 | 558406 | 634 | 347 | 685835 | 293750019 | 428.31 | 8949 | 102 |
| CNI_S3 | 46984914 | 46274596 | 98.49 | 373575 | 649 | 344 | 456722 | 197228166 | 431.83 | 9423 | 102 |
| CEU_S1 | 48395766 | 47634718 | 98.43 | 398303 | 551 | 339 | 467692 | 186808140 | 399.43 | 4146 | 102 |
| CEU_S2 | 47697886 | 47033300 | 98.6 | 351454 | 562 | 338 | 415242 | 168957756 | 406.89 | 6321 | 102 |
| CEU_S3 | 57849736 | 56939226 | 98.43 | 568535 | 528 | 335 | 662868 | 260186418 | 392.52 | 6663 | 102 |
| CTU_S1 | 54382854 | 53650758 | 98.65 | 504278 | 542 | 337 | 586393 | 230865864 | 393.71 | 6012 | 102 |
| CTU_S2 | 50255228 | 49538730 | 98.57 | 395313 | 559 | 339 | 467452 | 190053900 | 406.57 | 9627 | 102 |
| CTU_S3 | 49953534 | 49267802 | 98.63 | 471531 | 549 | 337 | 554213 | 219413775 | 395.9 | 4728 | 102 |
| CPA_S1 | 53797698 | 53110184 | 98.72 | 258421 | 486 | 331 | 292691 | 112119897 | 383.07 | 3891 | 102 |
| CPA_S2 | 47782278 | 47019746 | 98.40 | 223777 | 500 | 332 | 255333 | 98504538 | 385.79 | 3285 | 102 |
| CPA_S3 | 52635746 | 51907586 | 98.62 | 317552 | 502 | 333 | 366466 | 141506457 | 386.14 | 5094 | 102 |
| CPU_S1 | 54106326 | 53411034 | 98.71 | 403126 | 505 | 334 | 466486 | 180851778 | 387.69 | 3960 | 102 |
| CPU_S2 | 54572502 | 53800514 | 98.59 | 501608 | 520 | 337 | 586982 | 231776379 | 394.86 | 4161 | 102 |
| CPU_S3 | 45199128 | 44552892 | 98.57 | 348607 | 591 | 341 | 422081 | 175406670 | 415.58 | 4338 | 102 |
| CPE_S1 | 49228462 | 48576582 | 98.68 | 534918 | 590 | 343 | 649985 | 267951081 | 412.24 | 4722 | 102 |
| CPE_S2 | 51236696 | 50504388 | 98.57 | 435740 | 505 | 333 | 508501 | 196806294 | 387.03 | 5766 | 102 |
| CPE_S3 | 60937686 | 59874518 | 98.26 | 708381 | 616 | 345 | 878828 | 369167118 | 420.07 | 7839 | 102 |
| CGR_S1 | 51138252 | 50359076 | 98.48 | 648004 | 599 | 346 | 798233 | 332802591 | 416.92 | 6660 | 102 |
| CGR_S2 | 48526850 | 47739914 | 98.38 | 312469 | 492 | 331 | 359588 | 137613318 | 382.7 | 4878 | 102 |
| CGR_S3 | 50085004 | 49415288 | 98.66 | 442708 | 510 | 334 | 515331 | 201400683 | 390.82 | 7956 | 102 |
| CLI_S1 | 53725054 | 53005242 | 98.66 | 520853 | 551 | 339 | 617729 | 245892456 | 398.06 | 5235 | 102 |
| CLI_S2 | 48834682 | 48138110 | 98.57 | 358946 | 546 | 337 | 422545 | 168772854 | 399.42 | 5424 | 102 |
| CLI_S3 | 50368230 | 49620150 | 98.51 | 446622 | 546 | 338 | 525823 | 209534817 | 398.49 | 5751 | 102 |
