# Supplementary material for: Combined analysis of metagenome and transcriptome revealed the adaptive mechanism of different golden Camellia species in karst regions
Source: Front Plant Sci. 2023 Nov 20;14:1180472. doi: 10.3389/fpls.2023.1180472 (PMC10699447; doi:10.3389/fpls.2023.1180472)
Supplement: Supplementary file 3 [file Table_3.docx]

Table S3 Characteristics of RNA-sequencing results of different root samples

| Samples | Raw reads | Clean reads | Error rate(%) | Q20(%) | Q30(%) | GC content(%) | Total reads | Total mapped | Multiple mapped | Uniquely mapped |
| --- | --- | --- | --- | --- | --- | --- | --- | --- | --- | --- |
| CNI_R1 | 49755810 | 47596192 | 0.0266 | 97.4 | 92.61 | 44.86 | 47596192 | 35553942(74.7%) | 2212596(4.65%) | 33341346(70.05%) |
| CNI_R2 | 46397672 | 44559950 | 0.0262 | 97.58 | 93.09 | 45.37 | 44559950 | 31389330(70.44%) | 2104501(4.72%) | 29284829(65.72%) |
| CNI_R3 | 59929928 | 57705472 | 0.0266 | 97.45 | 92.76 | 44.67 | 57705472 | 40376351(69.97%) | 2854960(4.95%) | 37521391(65.02%) |
| CEU_R1 | 44352832 | 41917646 | 0.0269 | 97.26 | 92.44 | 46.81 | 41917646 | 29613040(70.65%) | 2261457(5.39%) | 27351583(65.25%) |
| CEU_R2 | 49067376 | 47308598 | 0.027 | 97.24 | 92.31 | 46.43 | 47308598 | 32686102(69.09%) | 2602015(5.5%) | 30084087(63.59%) |
| CEU_R3 | 47625708 | 45663738 | 0.0265 | 97.46 | 92.8 | 46.75 | 45663738 | 34566842(75.7%) | 2582332(5.66%) | 31984510(70.04%) |
| CTU_R1 | 45731512 | 43040434 | 0.0265 | 97.46 | 92.82 | 44.27 | 43040434 | 29464225(68.46%) | 2047918(4.76%) | 27416307(63.7%) |
| CTU_R2 | 53209628 | 49890166 | 0.0278 | 96.9 | 91.64 | 44.23 | 49890166 | 33934598(68.02%) | 2397838(4.81%) | 31536760(63.21%) |
| CTU_R3 | 41960128 | 39367652 | 0.0274 | 97.07 | 92.04 | 44.22 | 39367652 | 26878742(68.28%) | 1906145(4.84%) | 24972597(63.43%) |
| CPA_R1 | 57706412 | 56025718 | 0.0264 | 97.54 | 92.93 | 44.74 | 56025718 | 40141520(71.65%) | 3153289(5.63%) | 36988231(66.02%) |
| CPA_R2 | 55018916 | 52975568 | 0.0261 | 97.64 | 93.19 | 44.36 | 52975568 | 38439331(72.56%) | 3571346(6.74%) | 34867985(65.82%) |
| CPA_R3 | 48155954 | 45733626 | 0.0269 | 97.29 | 92.36 | 45.18 | 45733626 | 34103099(74.57%) | 2409651(5.27%) | 31693448(69.3%) |
| CPU_R1 | 54729728 | 53003320 | 0.026 | 97.67 | 93.25 | 44.47 | 53003320 | 39072015(73.72%) | 2737940(5.17%) | 36334075(68.55%) |
| CPU_R2 | 45786328 | 43715026 | 0.0261 | 97.63 | 93.18 | 44.6 | 43715026 | 31369249(71.76%) | 2264195(5.18%) | 29105054(66.58%) |
| CPU_R3 | 44106662 | 42736814 | 0.0264 | 97.51 | 92.85 | 44.45 | 42736814 | 31319882(73.29%) | 2240687(5.24%) | 29079195(68.04%) |
| CPE_R1 | 54893300 | 52837872 | 0.0259 | 97.7 | 93.36 | 44.41 | 52837872 | 38387028(72.65%) | 2553452(4.83%) | 35833576(67.82%) |
| CPE_R2 | 43694182 | 41990742 | 0.0259 | 97.71 | 93.37 | 44.08 | 41990742 | 29848713(71.08%) | 2102078(5.01%) | 27746635(66.08%) |
| CPE_R3 | 52156954 | 50201044 | 0.0265 | 97.48 | 92.81 | 44.6 | 50201044 | 36418471(72.55%) | 2578294(5.14%) | 33840177(67.41%) |
| CGR_R1 | 45434224 | 44481796 | 0.0262 | 97.65 | 92.99 | 46.05 | 44481796 | 33460261(75.22%) | 2717100(6.11%) | 30743161(69.11%) |
| CGR_R2 | 44601236 | 42441640 | 0.0265 | 97.44 | 92.78 | 45.97 | 42441640 | 32209504(75.89%) | 2459126(5.79%) | 29750378(70.1%) |
| CGR_R3 | 44717000 | 42390962 | 0.0261 | 97.59 | 93.08 | 45.58 | 42390962 | 32215409(76.0%) | 2391827(5.64%) | 29823582(70.35%) |
| CLI_R1 | 44288276 | 42395858 | 0.0261 | 97.6 | 93.12 | 44.99 | 42395858 | 31565358(74.45%) | 2618116(6.18%) | 28947242(68.28%) |
| CLI_R2 | 43296504 | 41108400 | 0.0271 | 97.2 | 92.29 | 47.64 | 41108400 | 31481855(76.58%) | 3503210(8.52%) | 27978645(68.06%) |
| CLI_R3 | 43307158 | 41494998 | 0.0261 | 97.62 | 93.17 | 44.99 | 41494998 | 30870097(74.39%) | 2319340(5.59%) | 28550757(68.81%) |
