# Supplementary material for: Combined analysis of metagenome and transcriptome revealed the adaptive mechanism of different golden Camellia species in karst regions
Source: Front Plant Sci. 2023 Nov 20;14:1180472. doi: 10.3389/fpls.2023.1180472 (PMC10699447; doi:10.3389/fpls.2023.1180472)
Supplement: Supplementary file 4 [file Table_4.docx]

Table S4 Annotation of soil rhizosphere microbial metagenes in KEGG database

| Level1 | Level2 | Cni | Ceu | Ctu | Cpa | Cpu | Cpe | Cgr | Cli | Total | |
| --- | --- | --- | --- | --- | --- | --- | --- | --- | --- | --- | --- |
| Cellular Processes | Cellular community - prokaryotes | 747268 | 671072 | 701512 | 461760 | 713910 | 1022434 | 869286 | 871076 | 6058318 | 9140904 |
|  | Cell motility | 80128 | 61552 | 66186 | 44268 | 56942 | 86898 | 66840 | 69696 | 532510 |  |
|  | Transport and catabolism | 104550 | 75030 | 79970 | 44318 | 80712 | 106394 | 86346 | 83200 | 660520 |  |
|  | Cell growth and death | 263910 | 232424 | 231632 | 138976 | 222286 | 307052 | 246198 | 245230 | 1887708 |  |
|  | Cellular community - eukaryotes | 240 | 150 | 188 | 126 | 456 | 224 | 258 | 206 | 1848 |  |
| Environmental Information Processing | Membrane transport | 552584 | 558088 | 583706 | 429760 | 674948 | 956624 | 780538 | 834928 | 5371176 | 9641608 |
|  | Signal transduction | 660582 | 450188 | 499780 | 303650 | 507540 | 698636 | 571586 | 576496.00 | 4268458 |  |
|  | Signaling molecules and interaction | 312 | 136 | 124 | 180 | 284 | 418 | 300 | 220.00 | 1974 |  |
| Genetic Information Processing | Folding, sorting and degradation | 216438 | 174364 | 183896 | 130108 | 221352 | 263652 | 216078 | 202796.00 | 1608684 | 6194344 |
|  | Transcription | 36622 | 33412 | 35636 | 34914 | 50736 | 49024 | 43232 | 42542.00 | 326118 |  |
|  | Replication and repair | 294588 | 234314 | 254538 | 147764 | 270856 | 352168 | 276832 | 254094.00 | 2085154 |  |
|  | Translation | 288062 | 228590 | 248448 | 174370 | 306138 | 355094 | 296854 | 276832.00 | 2174388 |  |
| Human Diseases | Infectious disease: viral | 15894 | 13140 | 13998 | 8028 | 14884 | 15810 | 12984 | 13468.00 | 108206 | 5246280 |
|  | Drug resistance: antineoplastic | 45898 | 43494 | 42792 | 22854 | 36098 | 53614 | 42064 | 45382.00 | 332196 |  |
|  | Substance dependence | 7982 | 7678 | 7772 | 3582 | 8604 | 11666 | 9164 | 7414.00 | 63862 |  |
|  | Cancer: overview | 106864 | 90594 | 91128 | 53554 | 87210 | 126408 | 96608 | 98840.00 | 751206 |  |
|  | Immune disease | 8216 | 4392 | 5002 | 2190 | 4472 | 6710 | 4886 | 3664.00 | 39532 |  |
|  | Drug resistance: antimicrobial | 204964 | 123972 | 145304 | 82588 | 143826 | 189574 | 152476 | 152078.00 | 1194782 |  |
|  | Infectious disease: parasitic | 26722 | 18508 | 18856 | 6858 | 13352 | 19792 | 14918 | 13038.00 | 132044 |  |
|  | Endocrine and metabolic disease | 89166 | 66048 | 67584 | 41368 | 66640 | 87942 | 69620 | 67674.00 | 556042 |  |
|  | Cancer: specific types | 36996 | 35940 | 34240 | 18072 | 24948 | 42582 | 31362 | 35354.00 | 259494 |  |
|  | Infectious disease: bacterial | 117578 | 87604 | 94054 | 61568 | 89950 | 116740 | 94984 | 89726.00 | 752204 |  |
|  | Neurodegenerative disease | 38736 | 32870 | 34414 | 21200 | 36956 | 46398 | 36966 | 38692.00 | 286232 |  |
|  | Cardiovascular disease | 105156 | 94192 | 94198 | 57820 | 91094 | 124780 | 99336 | 103904.00 | 770480 |  |
| Metabolism | Amino acid metabolism | 1246492 | 1031434 | 1078190 | 688082 | 1194188 | 1536526 | 1254894 | 1216258.00 | 9246064 | 77882796 |
|  | Energy metabolism | 937766 | 722060 | 769572 | 505048 | 823062 | 1062918 | 858432 | 837714.00 | 6516572 |  |
|  | Global and overview maps | 4426114 | 3432948 | 3656128 | 2155168 | 3728200 | 4974178 | 4003502 | 3897418.00 | 30273656 |  |
|  | Carbohydrate metabolism | 1551522 | 1135634 | 1220032 | 698480 | 1190624 | 1604850 | 1287682 | 1221808.00 | 9910632 |  |
|  | Glycan biosynthesis and metabolism | 341390 | 236282 | 266554 | 136252 | 256656 | 354632 | 280668 | 262774.00 | 2135208 |  |
|  | Nucleotide metabolism | 413816 | 345854 | 356996 | 221902 | 382250 | 505610 | 401832 | 381436.00 | 3009696 |  |
|  | Xenobiotics biodegradation and metabolism | 468302 | 404798 | 406928 | 243714 | 377600 | 529850 | 433666 | 463378.00 | 3328236 |  |
|  | Lipid metabolism | 418782 | 310688 | 321962 | 182030 | 314314 | 437064 | 348566 | 329478.00 | 2662884 |  |
|  | Metabolism of other amino acids | 399502 | 313022 | 330018 | 185194 | 320248 | 428682 | 341740 | 331332.00 | 2649738 |  |
|  | Metabolism of cofactors and vitamins | 720498 | 550284 | 588298 | 352602 | 640622 | 839792 | 675178 | 656216.00 | 5023490 |  |
|  | Metabolism of terpenoids and polyketides | 242148 | 165980 | 183368 | 93148 | 167604 | 240602 | 186940 | 167838.00 | 1447628 |  |
|  | Biosynthesis of other secondary metabolites | 258796 | 185950 | 203236 | 111296 | 207330 | 280484 | 224396 | 207504.00 | 1678992 |  |
| Organismal Systems | Endocrine system | 170032 | 121502 | 126224 | 69216 | 111124 | 162720 | 129106 | 122580.00 | 1012504 | 3449094 |
|  | Nervous system | 28842 | 27976 | 28774 | 18852 | 36992 | 45938 | 34842 | 30792.00 | 253008 |  |
|  | Circulatory system | 7936 | 4172 | 4518 | 2500 | 3614 | 4742 | 3826 | 4346.00 | 35654 |  |
|  | Environmental adaptation | 105374 | 70806 | 74972 | 40726 | 63380 | 89310 | 71864 | 71546.00 | 587978 |  |
|  | Development and regeneration | 5572 | 4548 | 4488 | 2920 | 6692 | 7898 | 6416 | 4884.00 | 43418 |  |
|  | Excretory system | 9444 | 7538 | 8568 | 6768 | 11106 | 12888 | 10482 | 8922.00 | 75716 |  |
|  | Aging | 149886 | 150946 | 142712 | 85056 | 125842 | 183078 | 146444 | 149532.00 | 1133496 |  |
|  | Digestive system | 37792 | 29366 | 29206 | 17952 | 32316 | 40728 | 34416 | 32708.00 | 254484 |  |
|  | Immune system | 5512 | 5798 | 5116 | 4238 | 7132 | 9870 | 7244 | 7490.00 | 52400 |  |
|  | Sensory system | 12 | 38 | 116 | 14 | 96 | 102 | 40 | 18.00 | 436 |  |
