# Supplementary material for: Combined analysis of metagenome and transcriptome revealed the adaptive mechanism of different golden Camellia species in karst regions
Source: Front Plant Sci. 2023 Nov 20;14:1180472. doi: 10.3389/fpls.2023.1180472 (PMC10699447; doi:10.3389/fpls.2023.1180472)
Supplement: Supplementary file 5 [file Table_5.docx]

Table S5 Annotation of soil rhizosphere microbial metagenes in CAZy database

| Class | Cni | Ceu | Ctu | Cpa | Cpu | Cpe | Cgr | Cli | total |
| --- | --- | --- | --- | --- | --- | --- | --- | --- | --- |
| AA | 153054 | 95380 | 108790 | 50044 | 90874 | 144578 | 110552 | 101088 | 854360 |
| CBM | 22998 | 14296 | 17182 | 9770 | 18784 | 25556 | 19758 | 17764 | 146108 |
| CE | 212558 | 142916 | 165120 | 74662 | 132900 | 196230 | 156700 | 162128 | 1243214 |
| GH | 381792 | 221970 | 263904 | 103480 | 197168 | 305138 | 236344 | 212800 | 1922596 |
| GT | 446128 | 256310 | 303486 | 132944 | 244640 | 396124 | 312996 | 293898 | 2386526 |
| PL | 32428 | 14478 | 19500 | 9078 | 20444 | 27778 | 23450 | 21710 | 168866 |
