# Supplementary figures and images for: Combined analysis of metagenome and transcriptome revealed the adaptive mechanism of different golden Camellia species in karst regions
Source: Front Plant Sci. 2023 Nov 20;14:1180472. doi: 10.3389/fpls.2023.1180472 (PMC10699447; doi:10.3389/fpls.2023.1180472)

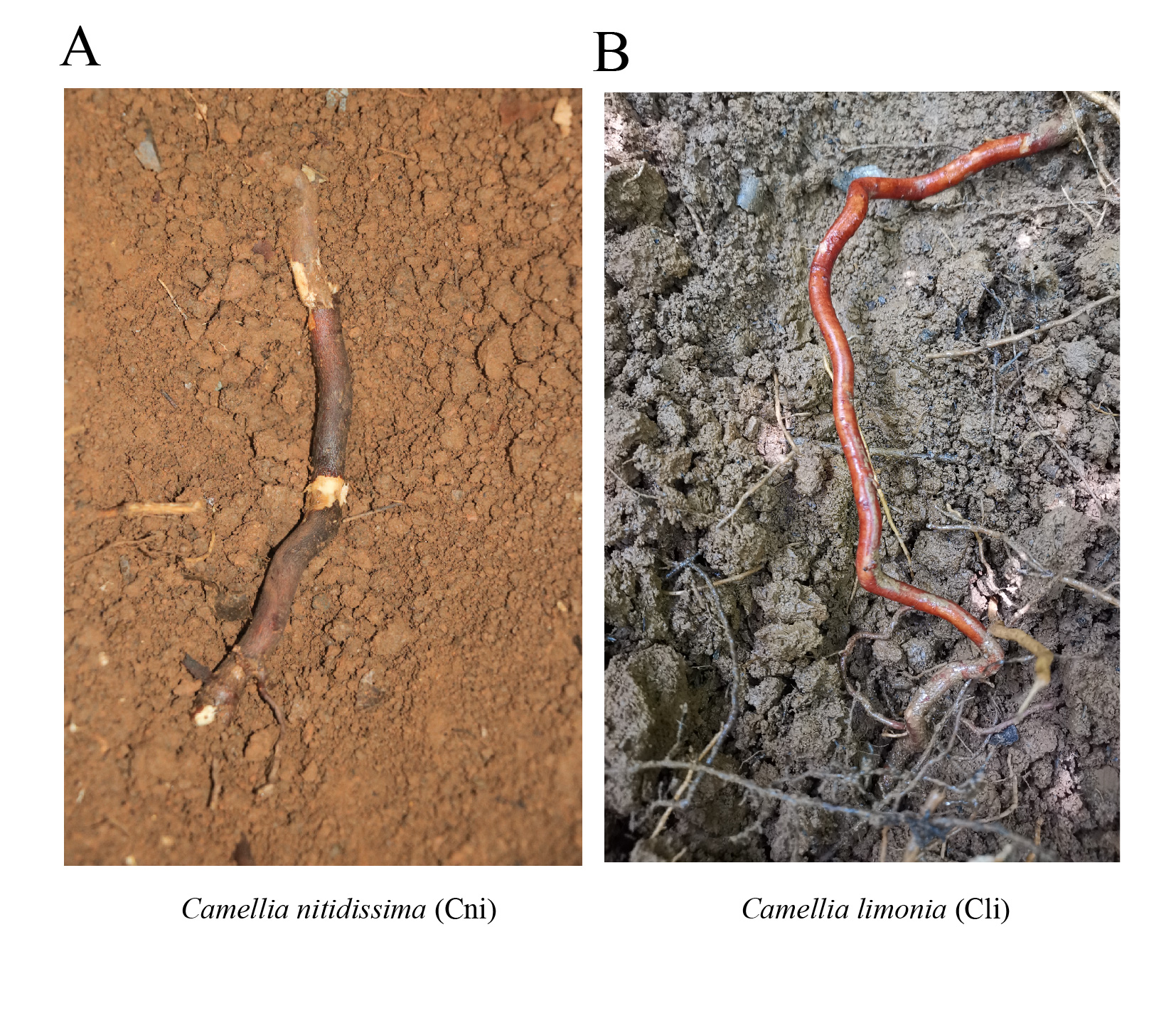

Supplement: Supplementary Figure 1 — Picture of sampling of golden Camellia growing in acidic soil and karst soil. (A) Sampling of Cni. (B) Sampling of Cli. [file Image_1.jpeg]

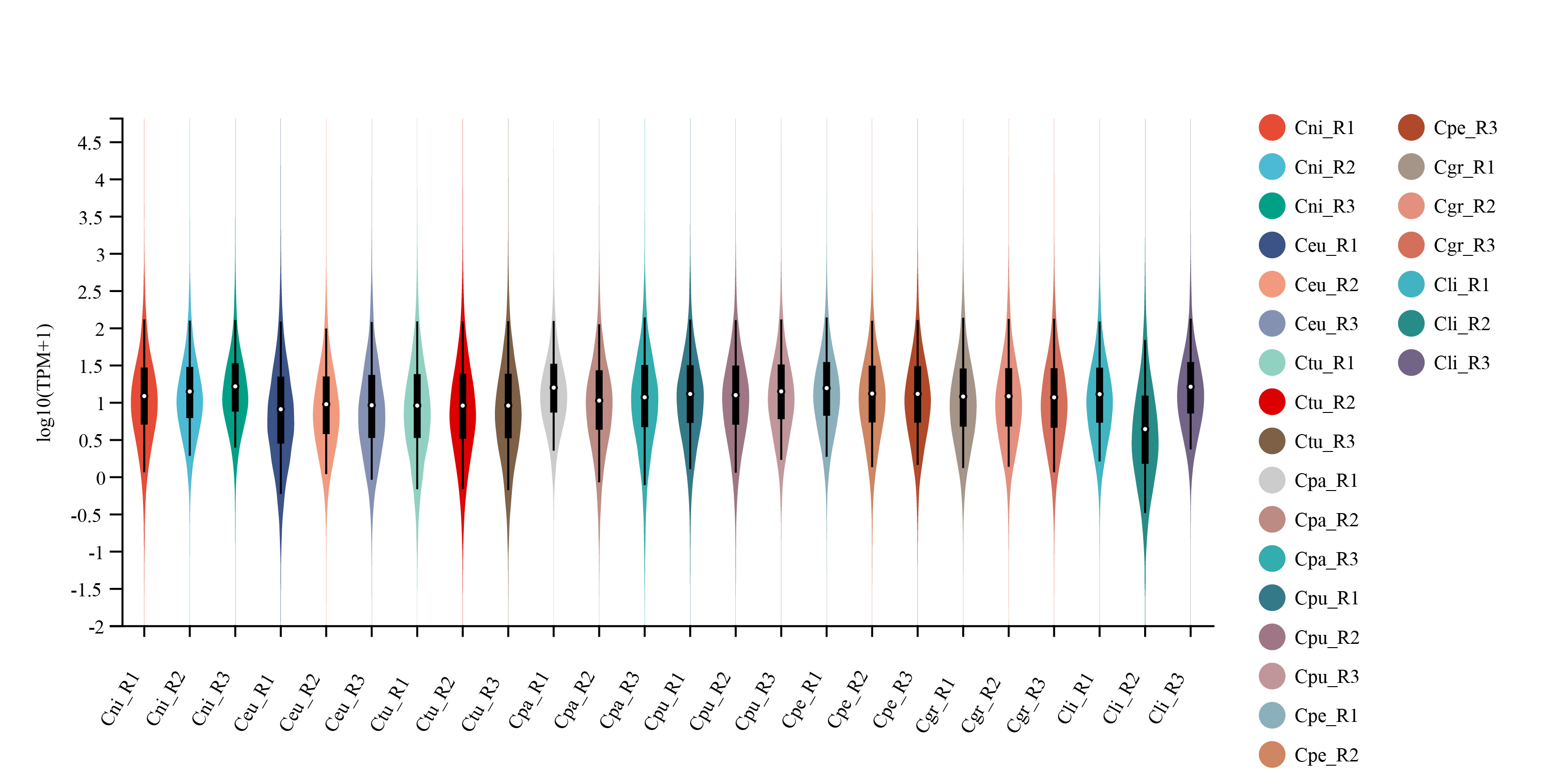

Supplement: Supplementary Figure 2 — Expression level of each root sample. The quantitative index was TPM. [file Image_2.jpeg]

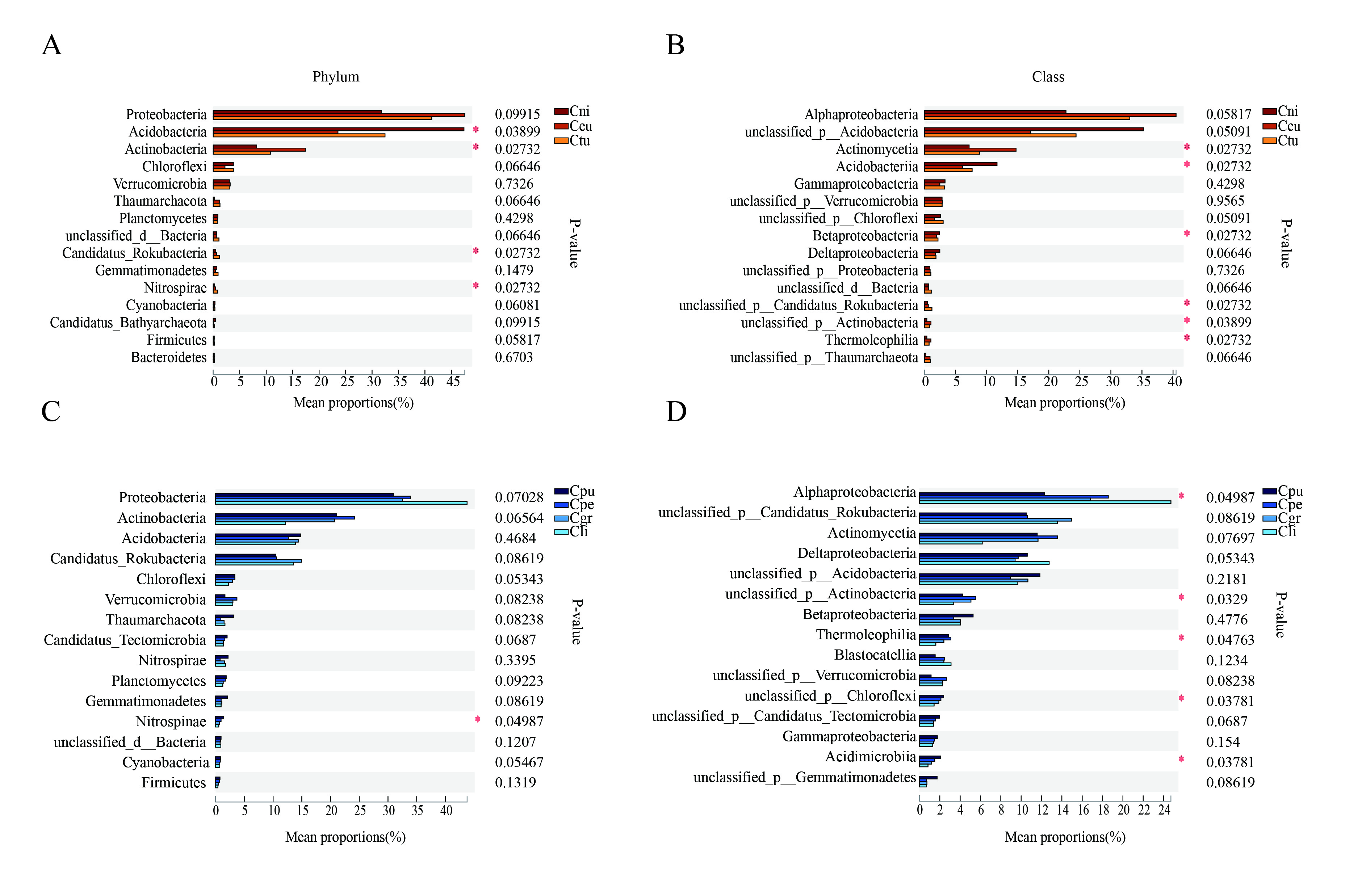

Supplement: Supplementary Figure 3 — Differences in rhizosphere microbial community composition. (A) Between Cni, Ceu, Ctu, and Cpa at the phylum level. (B) Between Cni, Ceu, Ctu, and Cpa at the class level. (C) Between Cpu, Cpe, Cgr, and Cli at the phylum level. (D) Between Cpu, Cpe, Cgr, and Cli at the phylum level and class level. [file Image_3.jpeg]

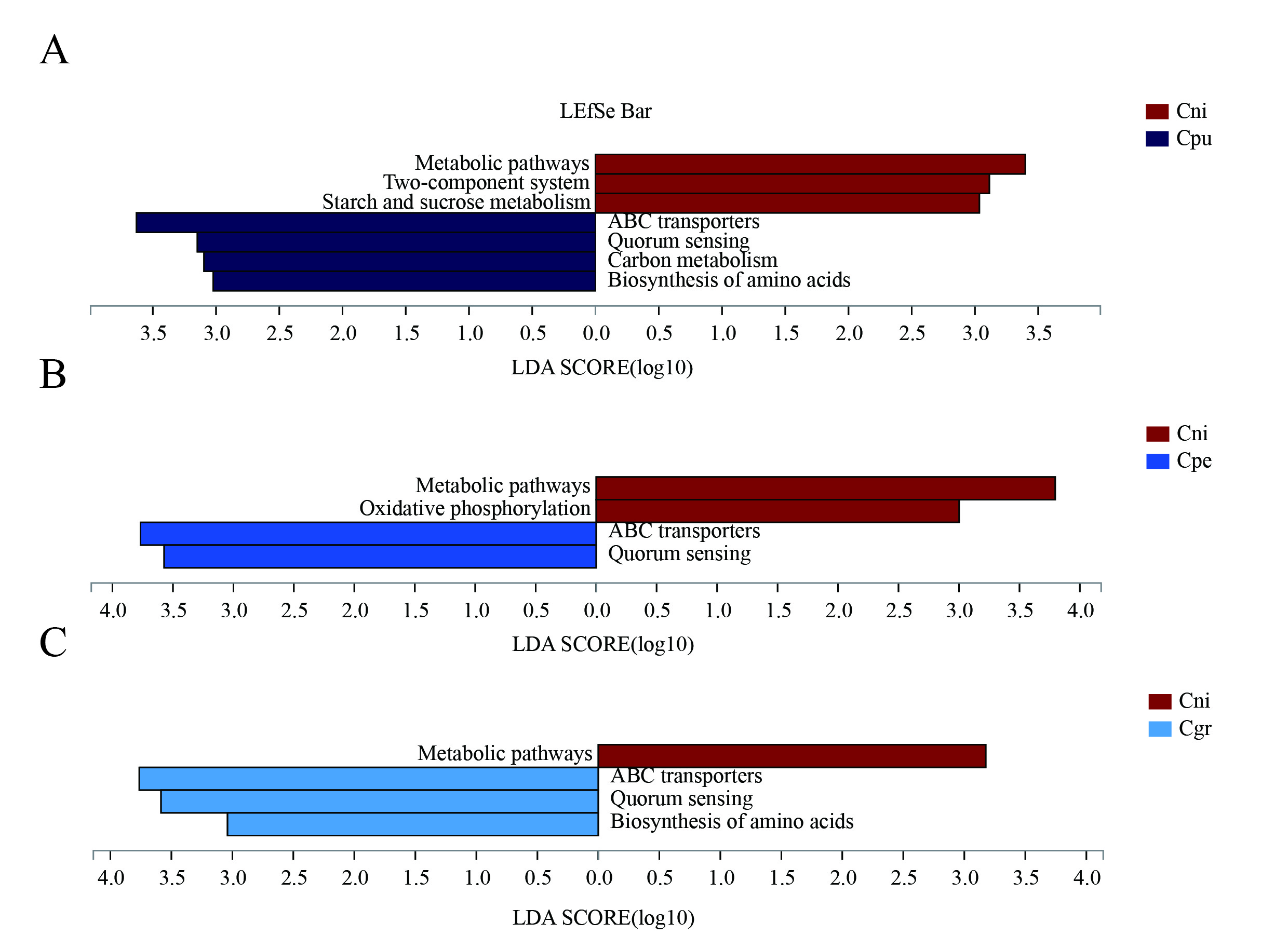

Supplement: Supplementary Figure 4 — Gene functions of rhizosphere microbial communities. (A) LEfSe analysis at KEGG pathway level 3 with significant differences in Cni and Cpu. (B) In Cni and Cpe. (C) In Cni and Cgr. [file Image_4.jpeg]

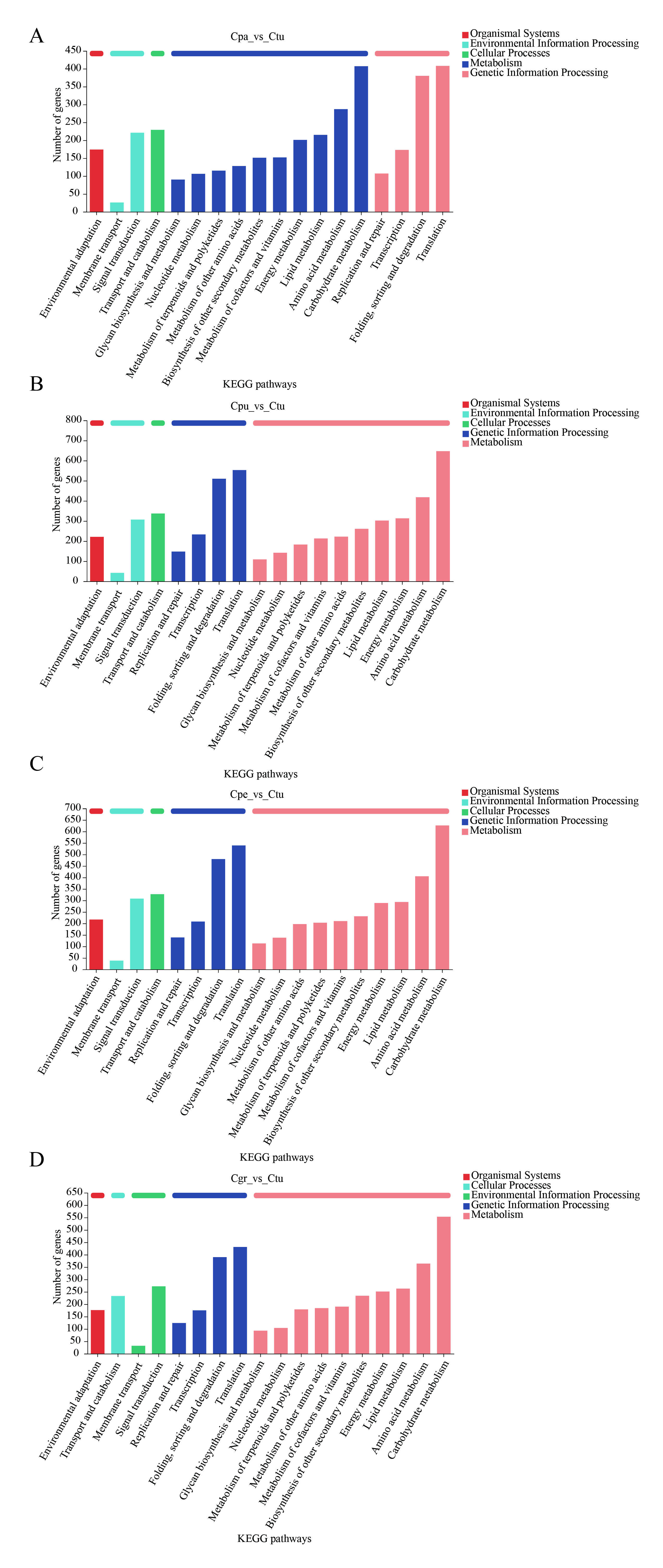

Supplement: Supplementary Figure 5 — Functional annotation of DEGs based on the KEGG database. (A) In Cpa_vs_Ctu. (B) In Cpu_vs_Ctu. (C) In Cpe_vs_Ctu. (D) In Cgr_vs_Ctu. [file Image_5.jpeg]
